# Controlling the harmonic generation in transition metal dichalcogenides and their heterostructures

**DOI:** 10.1515/nanoph-2022-0159

**Published:** 2022-04-26

**Authors:** Renlong Zhou, Alex Krasnok, Naveed Hussain, Sa Yang, Kaleem Ullah

**Affiliations:** School of Physics and Information Engineering, Guangdong University of Education, No. 351 Xinggang Road, Guangzhou, 510303, P. R. China; Department of Electrical and Computer Engineering, Florida International University, Miami, FL 33174, USA; Institute of Fundamental and Frontier Sciences, University of Electronic Science and Technology of China, Chengdu 610054, Sichuan, P. R. China; School of Electronic Science and Engineering, Nanjing University, Nanjing 210093, China

**Keywords:** enhancement, harmonic generation, heterostructure, modulation, transition metal dichalcogenides, twisting

## Abstract

The growing interest in transition metal dichalcogenides (TMDs) has encouraged researchers to focus on their nonlinear optical properties, such as harmonic generation (HG), which has potential for fundamental science and applications. HG is a nonlinear phenomenon used to study low-dimensional physics and has applications in bioimaging, optical signal processing, and novel coherent light sources. In this review, we present the state-of-the-art advances of HG in atomically-thin TMDs and their heterostructures. Different factors affecting the HG in TMDs such as strain, electric gating, excitonic resonance, phase and edge modulation, and valley-induced HG are discussed with a particular emphasis on the HG in heterostructure van der Waals TMDs. Moreover, we discuss the enhancement of HG in TMDs by incorporating cavities and nanostructures including the bound states in the continuum with extreme Q-factor. This work provides a concise summary of recent progress in engineering HG in atomically-thin TMDs and their heterostructures and a compact reference for researchers entering the field.

## 1 Introduction

Frequency conversion processes, such as second- and third-harmonic generation (HG), are among the most common effects in nonlinear optics, which offer many opportunities for photonics, material science, and characterization [1]. The study of these interactions is the essence of nonlinear optics, one of the bedrocks of modern photonics. In particular, the high sensitivity of harmonic generation (HG) on material properties, crystallinity, defects, and dependence on parameters of excitation enables a broad range of applications and nonlinear optical devices [2], [3], [4], [5]. Conventional nonlinear devices are made from bulk materials such as beta barium borate (BBO), potassium titanyl phosphate (KTP), or lithium niobate (LiNbO_3_). These materials are unsuitable for advanced technology demands, such as multi-functional or tunable on-chip devices [6, 7]. Plasmonic materials have been introduced as nonlinear materials since they can enhance the nonlinear processes up to several orders of magnitude by confining the field into a nanometric region [8], [9], [10], [11]. However, they are impractical for nonlinear materials due to high dissipative losses [12]. High-index dielectric nanomaterials have been introduced as a favorable alternative because of their strong magnetic response and low losses [13, 14]. Despite this progress, the efficiency of nonlinear effects in dielectric materials does not meet the requirements of modern nonlinear devices [6].

For more than a decade, two-dimensional (2D) materials [15] have been a hot topic owing to their unique material features, ultra-thin scale, and outstanding linear and nonlinear optical capabilities that make them ideal for a wide variety of device applications [16], [17], [18], [19], [20], [21], [22], [23], [24], [25], [26], [27], [28], [29], [30], [31], [32], [33], [34], [35], [36], [37], [38], [39], [40], [41], [42], [43], [44], [45], [46], [47]. After discovering graphene in 2004 [48], many efforts have been made to uncover the HG physics in 2D materials [2, 3, 5, 49, 50]. In the last decade, transition metal dichalcogenides (TMDs) have garnered significant attention because of their unique physical properties, such as high exciton binding energies [51], [52], [53], direct bandgap in the visible and IR ranges [54], [55], [56], [57], and naturally occurring intrinsic valley polarization [58], [59], [60]. Around 2013, researchers began to investigate HG in TMDs [7, 49, 61, 62]. The research of harmonic generation is also conducted in various 2D materials. For example, graphene [63], black phosphorus [64], and other 2D materials [50] had equivalent HG qualities to TMDs and exhibited comparable *χ* ^(2)^ and *χ* ^(3)^ [50]. However, HG research on TMDs attracted the attention of many researchers due to the following considerations. First, their typical binary crystal structure allows breaking inversion symmetry enabling even-order HG processes, which is not the case in graphene [65]. Second, there are numerous possibilities for TMD homo- and heterostructures configurations with strong and tunable HG responses [66]. Last but not least, HG in TMDs is a rapidly developing area with a lot of novel physics, such as valley selective second harmonic generation (SHG) [67], controlling the HG with a twist [68], Moiré nanostructures, polaritons [69], to name a few. Furthermore, it has been demonstrated that the excitonic states in TMDs are highly correlated with the HG response delivered by TMDs and their heterostructures [70, 71]. One of the recent trends is to use the HG as a tool to determine the twisting angle in homo- and heterostructure TMDs and to find the individual contribution of each layer to the total HG [68, 72], [73], [74], [75], [76], [77], [78], [79], [80], [81], [82], [83], [84], [85], [86]. The integration of resonant nanostructures with TMDs has also attracted a great deal of attention as they boost the HG from TMDs [69, 87], [88], [89], [90], [91], [92], [93], [94], [95], [96], [97], [98], [99], [100], [101], [102], [103], [104], [105], [106], [107], [108], [109], [110], [111], [112]. In addition, several other factors, including strain [113], electric gating [67], excitonic resonance [114], unusual optical resonances [115], phase and edge modulation [77, 116, 117], etc., also have been used to manipulate the HG in TMDs.

In this work, we present the progress in the field of HG in TMDs and their heterostructures. We discuss various operations available to set up or control the HG in TMDs. Note that there are a number of reviews [2, 3, 5, 7] addressing different particular aspects of this research area. However, these reviews do not present several important aspects of HG in TMDs emerged recently, including HG in van der Waals homo- and heterostructure TMDs. The rest of the work is as follows. Section 2 discusses the theoretical basis of HG in TMDs. Various operations are presented in Sections 3 and 4 regarding the manipulation or tuning of the HG in TMDs, with a particular emphasis on twisted and heterostructure TMDs. In conclusion, we provide a perspective and outlook of this actively developing research area.

## 2 Basics

Light propagation in free space is linear as nonlinearity requires the presence of matter and strong intensity [7]. In the weak intensity regime, materials respond linearly to applied electric fields E(r,t) :

(1) $$P_{\text{L}}\left(r,t\right)=\epsilon_{\text{o}}\left(\chi^{\left(1\right)}E\left(r,t\right)\right).$$

Here, *P*_L_(*r*, *t*) is the linear polarization, *χ* ^(1)^ is the linear susceptibility, *ϵ*_o_ is the permittivity of the free space, and *E*(*r*, *t*) is the electric field of light. Microscopically, the optical nonlinearity in a material can only be observed when the electric field of the impinging light is comparable to the interatomic electric field, typically ∼10^5^–10^8^ V/m [7]. Therefore, for realizing optical nonlinearity, a powerful light source is necessary to trigger the nonlinear optical response in the material under study.

With the invention of the laser, the first nonlinear optical response was observed in 1961 [118] for intense light in quartz. The nonlinearity gives rise to the nonlinear polarization *P*_NL_(*r*, *t*),

(2) $$P_{\text{NL}}\left(r,t\right)=\epsilon_{\text{o}}\left(\chi^{\left(2\right)}E{\left(r,t\right)}^{2}+\chi^{\left(3\right)}E{\left(r,t\right)}^{3}+\ldots +\chi^{\left(n\right)}E\left(r,t^{n}\right)\right).$$

The *χ* ^(*n*)^ represents the nonlinear susceptibility of the *n*th order. The nonlinear susceptibility *χ* ^*n*^ is used to characterize the material’s capacity to excite the nonlinear processes where “*n*” denotes the order of the nonlinear process. The real part of *χ* ^*n*^ represents the optical HG, whereas the imaginary part is responsible for the multiphoton resonance, optical limiting, and saturable absorption [49]. For example, the second-order nonlinearity is a tensor of rank 3 and can be characterized by the second-order susceptibility *χ* ^(2)^ [1, 119]. The *χ* ^(2)^ is responsible for three-wave mixing, which means that two waves having frequencies *ω*_1_ and *ω*_2_ interact inside the nonlinear material to give rise to sum–frequency (*ω*_3_ = *ω*_1_ + *ω*_2_) or the difference frequency (*ω*_3_ = *ω*_1_ − *ω*_2_). Second-harmonic generation (SHG) is a degenerate case of sum–frequency with *ω*_1_ = *ω*_2_. The term *χ* ^(3)^ is responsible for the third harmonic generation (THG), four-wave mixing, self-phase modulation, self-phase modulation, and optical Kerr effects. High-order HGs are challenging to excite compared to the lower-order harmonics. That is why SHG and THG are the most common HG processes. Note that this list is by no means complete, and many other phenomena can arise in the nonlinear regime [1]. Unlike linear effects, which are always proportional to the incident field, higher-order nonlinear phenomena are also subject to symmetry constraints. For instance, the first term in Eq. (2) becomes zero if the structure is centrosymmetric [1]. This fact is of fundamental importance since it explicitly forbids bulk crystals with centrosymmetric lattices to exhibit second-order nonlinear responses regardless of the intensity of the applied field.

Frequency conversion in various materials has been a subject of extensive research for many years. Very recent applications include nanostructured materials characterization [120], coherent ultraviolet light generation [121], supercontinuum white light generation [122], bioimaging and nanomedicine [123], quantum optics [124, 125], and broadening of the spectral range accessible with existing lasers. Building upon significant achievements in nonlinear optics based on conventional bulk materials, the modern research trend has shifted towards the miniaturization of nonlinear optical components placed in more compact setups embracing 2D materials.

**Figure 1:**
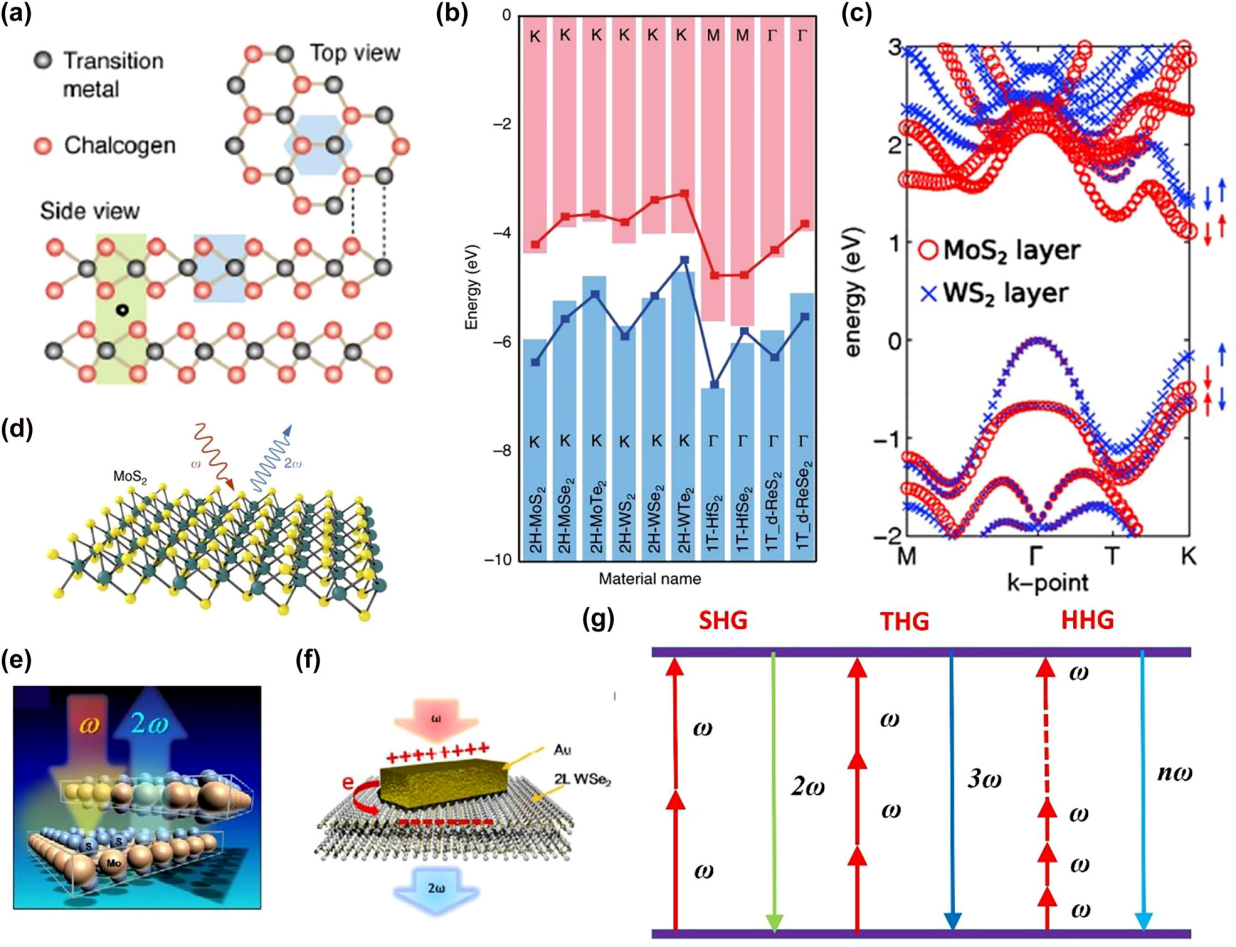
HG and band structure of TMDs. (a) Lattice structure of 2H-TMDs. The light green (blue) shaded region represents the unit cell of the 2L- (1L-) TMDs. (b) Calculated band alignments of various 1L-TMDs presented by the bar and line-point plots showing the conduction band minimum and valence band maximum on the Brillouin zone. (c) Calculated electronic band structure in a MoS_2_/WS_2_ heterostructure. The spin orientations of the wave functions at the *K*/*K*ʹ also indicated. (d)–(f) Schematic illustration of HG in (d) monolayer, (e) vdW and (f) nanostructure-TMDs structures. (g) Multi-photon processes: SHG (2*ω*), THG (3*ω*), and HHG (*nω*). The figures are reproduced with permission: (a) ref. [144] © 2017 American Chemical Society (b) ref. [145] © 2016 American Physical Society (c) ref. [146] © 2013 American Physical Society (d) ref. [113] © 2018 Nature Publishing Group (e) ref. [68] © 2014 American Chemical Society (f) ref. [97] © 2018 American Chemical Society.

The direct energy gap in TMDs makes them an excellent complement to graphene [126]. TMDs exhibit feeble van der Waals (vdW) forces between the layers, and their primary type (group VI semiconductors) is MX_2_ where M = W or Mo, and X = S, Se, Te. Each metal atom in TMDs is linked to six X atoms. These atoms are arranged hexagonally (Figure 1) and sandwiched by two X atoms to form a single layer. The monolayer (1L) is composed of covalent bonds between the atoms, while the bulk crystal is built by stacking monolayers with vdW forces between them [127]. The majority of TMD crystals are found in phase 2H [128]. When TMDs with 2H stacking are viewed as bulk crystals, their inversion symmetry belongs to the D_6h_ space group but changes from the D_6h_ to the D_3h_ space group when the symmetry breaks down [129]. Typically, thin layered TMDs in laboratory are manufactured using three processes including mechanical/liquid-phase exfoliation, molecular beam epitaxy (MBE) or chemical vapor deposition (CVD). Exfoliation is a rapid method for obtaining samples of TMDs with a few microns in diameter, which has aided in the research of HG in TMDs [130]. However, exfoliation is not appropriate for wide-area device manufacturing due to the unpredictability of manual operation, which makes it difficult to cover huge regions with a single flake or to control the shape or location of flakes. Additional transfer processes are often required to fabricate heterostructures of exfoliated TMDs [49]. In recent years, as the CVD process has improved, it has been effectively employed to create a variety of twisted TMDs. It is a widely used and viable approach for producing high-quality TMDs [66]. By regulating the gas flow and pressure of the gas, the researchers were able to create a variety of large area twisted 2D materials [130], [131], [132]. In comparison to other processes, CVD offers the benefit of high yield and the ability to meet the experimental requirements for twisted 2D materials with a variety of stacking orientations. Nonetheless, as compared to mechanical exfoliation, the quality of CVD-prepared TMDs is much worse [130]. Additionally, the CVD process is incapable of accurately controlling the angle of rotation between two monolayers of the 2D material. All of these characteristics significantly restrict the CVD technique’s use to the creation of twisted 2D materials. MBE is an advantageous technique for basic investigations of TMDs and their combinations due to its capacity to produce different forms of vertical heterostructures of 2D materials [132, 133]. Still, much work is necessary in MBE to improve the electrical characteristics of as-grown films, particularly since it is difficult to match the orientation of the overlayer with the lattice structure of the substrate, resulting in polycrystalline films with many dislocations [49].

vdW heterostructures made by stacking 1L-TMDs exhibit strong light–matter interactions, fast interlayer charge transfer, and valley-dependent selection rules [134]. As a result of weak vdW forces, the layers are stacked with no inter-diffusion of atoms or changes in lattice parameters. The resulting moiré superlattices demonstrate the novel properties [135]. As a result of the charge transfer between the 1L-TMDs, the interlayer excitons are formed, i.e., the electron and hole pairs in the different layers [136, 137]. These interlayer excitons possess high binding energies and interesting spin valley properties [138]. The strength of the interlayer interaction depends on the vertical distance between monolayers and their rotational alignments [139, 140]. This can be determined by their electronic band structure [135]. Unlike monolayer, the quenched photoluminescence (PL) of direct excitons was the first indication of the interlayer coupling within a TMD heterostructure [49, 141, 142]. After the PL spectroscopy, Raman spectroscopy was employed to detect the strong interlayer coupling by observing the breathing phonon modes in TMDs heterostructure [143]. In vdW TMDs, HG is a powerful and widely used tool for accurately measuring stacking order and twisting angle.

## 3 HG in TMDs

SHG is one of the most studied nonlinear optical processes in TMDs. A study conducted in 1998 by Wagoner et al. [147] examined bulk (3 µm) MoS_2_ and found a value of *χ* ^(2)^ for both 3R and 2H-MoS_2_ as large as ∼ (5 ± 2) × 10^−10^ and 5 × 10^−14^ mV^−1^, respectively. Later, Kumar et al. [61] in 2013 recorded high SHG from 1L-MoS_2_ samples and reported the *χ* ^(2)^ of ∼10^−9^ and 10^−7^ mV^−1^ for the CVD and exfoliated 1L-MoS_2_, respectively. This value is greater than the commercially used nonlinear materials such as BBO, KTP, and LiNbO_3,_ whose *χ* ^2^ is at the order of ∼10^−12^ mV^−1^ [7]. After that, several groups investigated the SHG in MoS_2_, WS_2_, MoSe_2_, WSe_2_, and MoTe_2_ [65, 77, 148], [149], [150], [151], [152], [153]. A comparison of *χ* ^2^ in these TMDs under the specific conditions is given in Table 1. For an accurate comparison of *χ* ^(2)^, one must consider several factors: working wavelength, fabrication method, substrate, thickness, etc. These all have a significant impact on the *χ* ^(2)^. These all parameters are included in Table 1. Inversion symmetry breaking is responsible for the larger *χ* ^(2)^ value of 1L-MoS_2_. In MoS_2_, it was found that SHG is detected only for an odd number of layers, while it can be neglected for an even number of layers. The surface effect ascribed diminishing SHG in an even number of layers. Although some studies report the SHG decreases when the odd number of layers increases from 1 to 5 [105], in other investigations, the SHG remains unchanged [154]. A more significant difference (four to five orders) was observed in the *χ* ^(2)^ values of TMDs. For instance, in the case of 1L-MoS_2_, the value is in the range of 10^−7^ to 10^−12^ mV^−1^ [61, 65]. The reason for such a vast difference in *χ* ^(2)^ may be attributed to several factors. The first factor is the fabrication method. For example, Kumar et al. [61] recorded the high differences in *χ* ^(2)^ between the exfoliated 1L-MoS_2_ and its CVD counterpart. The lower *χ* ^(2)^ (10^−7^ mV^−1^) in CVD MoS_2_ may be due to poor sample quality. Secondly, the polarization, well-defined in bulk materials, becomes worse defined in atomically thin layers [7]. Consequently, a greater deviation can be found in monolayer samples when using different estimation methodologies. Finally, the substrates can also affect the *χ* ^(2)^ in TMDs. The decrease in Si/SiO_2_ thickness from 270 to 125 nm resulted in several folds of SHG enhancement in 1L-MoS_2_ [155]. Changing interference from destructive (270 nm) to constructive (125 nm) caused the increase in SHG. SHG in 1L-TMD is also strongly affected by the polarization of the incoming laser beam and analyzer. A six-fold SHG pattern is formed when a laser beam with specific polarization is incident on a detector with a fixed analyzer in an optical path [156]. Polarization-dependent SHG is now known to be the fastest and most accurate method for determining crystal orientation in TMDs, particularly for TMD heterostructures. The HG in TMDs can be effectively controlled by various other pathways discussed in Section 5.

**Table 1:** A comparison of nonlinear optical susceptibilities (*χ* ^(2)^, *χ* ^(3)^) between various TMDs.

Name of TMDs	HG order	FW	Fabrication	Thickness	Substrate	*χ* ^(2)^ (m V^−1^)	Reference
						*χ* ^(3)^ (m^2^ V^−2^)	
MoS_2_	SHG	810 nm	ME	1L	SiO_2_/Si	∼10^−7^ mV^−1^	Ref [61]
		810 nm	CVD	1L	SiO_2_/Si	∼5 × 10^−9^ mV^−1^	Ref [61]
		1160 nm	CVD	1L	SiO_2_/Si	4.3 × 10^−10^ mV^−1^	Ref [169]
		1600 nm	CVD	1L	Fused SiO2	6.3 × 10^−12^ mV^−1^	Ref [170]
		1600 nm	CVD	1L	PET	6.3 × 10^−12^ mV^−1^	Ref [170]
		800 nm	ME	FL	SiO_2_/Si	1.4 × 10^−9^ mV^−1^	Ref [171]
		∼885 nm	ME	1L	Quartz	2.8 × 10^−9^ mV^−1^	Ref [148]
		1200 nm	ME	1L	SiO_2_/Si	4.05 × 10^−10^ mV^−1^	Ref [116]
		1560 nm	ME	1L	SiO_2_/Si	5.4 × 10^−12^ mV^−1^	Ref [65]
WS_2_		832 nm	CVD	1L	SiO_2_/Si	4.5 × 10^−9^ mV^−1^	Ref [172]
		1064 nm	LE	1L	Quartz	4.60 × 10^−10^ mV^−1^	Ref [173]
		∼1032 nm	ME	1L	SiO_2_/Si	∼1.6 × 10^−9^ mV^−1^	Ref [174]
		1560 nm	ME	1L	SiO_2_/Si	1.6 × 10^−11^ mV^−1^	Ref [65]
MoSe_2_		1620 nm	PLD + selenization	1L	SiO_2_/Si	5.0 × 10^−11^ mV^−1^	Ref [175]
		1560 nm	ME	1L	SiO_2_/Si	3.7 × 10^−11^ mV^−1^	Ref [65]
WSe_2_		∼885 nm	ME	1L	SiO_2_/Si	∼1.0 × 10^−9^ mV^−1^	Ref [174]
		∼1480 nm	ME	1L	SiO_2_/Si	∼6 × 10^−11^ mV^−1^	Ref [67]
		∼1550 nm	ME	1L	Fused SiO2	∼1 × 10^−10^ mV^−1^	Ref [176]
WSe_2_	SHG	1560 nm	ME	1L	SiO_2_/Si	1.6 × 10^−11^ mV^−1^	Ref [65]
		816 nm	ME	1L	SiO_2_/Si	5.0 × 10^−9^ mV^−1^	Ref [177]
MoTe_2_		992 nm	ME	FL	SiO_2_/Si	∼1.2 × 10^−9^ mV^−1^	Ref [178]
		1560 nm	ME	1L	SiO_2_/Si	2.5 × 10^−9^ mV^−1^	Ref [153]
MoS_2_	THG	1560 nm	CVD	1L	Glass	1.5 × 10^−19^ m^2^V^−2^	Ref [158]
		1758 nm	ME	FL	SiO_2_/Si	1.0 × 10^−19^ m^2^V^−2^	Ref [157]
		1560 nm	CVD	1L	SiO_2_/Si	1.2 × 10^−19^ m^2^V^−2^	Ref [149]
		1560 nm	ME	1L	SiO_2_/Si	3.6 × 10^−19^ m^2^V^−2^	Ref [65]
WS_2_		1560 nm	ME	1L	SiO_2_/Si	2.4 × 10^−19^ m^2^V^−2^	Ref [65]
MoSe_2_		1560 nm	ME	1L	SiO_2_/Si	2.2 × 10^−19^ m^2^V^−2^	Ref [65]
WSe_2_		∼1550 nm	ME	1L	Fused SiO2	∼1.4 × 10^−19^ m^2^V^−2^	Ref [176]
		1560 nm	ME	1L	SiO_2_/Si	1.0 × 10^−19^ m^2^V^−2^	Ref [65]

FW, fundamental wavelength; ME, mechanical exfoliation; LE, liquid exfoliation; CVD, chemical vapour deposition; PVD, physical vapour deposition; PLD, pulsed laser deposition; 1L, monolayer; FL, few layer.

**Table 2:** The key conditions for the HG in vdW TMDs.

TMDs based heterostructure	HG order	F.W	Fabrication	Enhancement/modulation/other	Reference
Twisted 2L-MoS_2_, WSe_2_/MoS_2_, WSe_2_/WS_2_	SHG	810 nm	CVD and transfer	The SHG from the twisted bilayers is a coherent superposition of the SHG fields from the individual layers, with a phase difference depending on the stacking angle	Ref [68]
Twisted 2L-MoS_2_	SHG	810 nm	CVD and transfer	Tuning of SHG was observed from maximum to minimum when changing the twisting angle from 0 to 60 degrees, respectively	Ref [82]
Lateral MoS_2_–WSe_2_ p–n heterojunctions	SHG	870 nm	Two steps CVD	No modulation in SHG across the junction, which suggests that the MoS_2_ grew out from the edges of WSe_2_ without misorientation	Ref [79]
MoS_2_ 2L and 3L stacks	SHG	830 nm	ME and transfer	Layer-dependent SHG in 2L and 3L stacking of MoS_2_. The maximum SHG intensity for the 2L-MoS_2_ is observed for a stacking angle of 28°	Ref [85]
Few-layered MoS_2_–graphene	SHG	800 nm	CVD and transfer	A strongly suppressed SHG from the MoS_2_–graphene heterostructure was recorded due to the strong interlayer coupling between the MoS_2_ and graphene layers	Ref [86]
MoS_2_ vertical/planar spiral nanosheets	SHG	780 nm	Direct CVD	Relatively stronger SHG was found in vertical and planar spiral MoS_2_ as compared to 1L-MoS_2_	Ref [81]
		1550 nm			
MoS_2_ stacked layers	SHG	680–1300 nm	Direct APCVD	MoS2 1L, 2L-AA and 3L-AAA showed increased SHG with an increasing thickness due to the cumulative nature of the broken inversion symmetry, whereas the 2L-AB crystal showed weak SHG due to restored inversion symmetry	Ref [74]
Few-layered MoS_2_–WS_2_	SHG	795 nm	ME and transfer	The space-charge field was created which induces an additional SHG (field-induced SHG) in the heterostructure	Ref [73]
Vertical MoS_2-_ MoS_2(1−*x*)_Se_2*x*_	SHG	1120–1500 nm	CVD and transfer	By modulating the interlayer coupling with the fabrication of different kinds of hetero bilayers, the SHG can be significantly controlled	Ref [76]
MoS_2_–WS_2_	Valley-dependent SHG	800 nm	Two steps CVD	The interlayer coupling has no significant effect on valley-dependent SHG. Valley-dependent SHG polarization increases by decreasing temperature because of suppression of the interlayer scattering	Ref [83]
MoS_2_ vertical/planar spiral nanosheets	THG	1550 nm	Direct CVD	Relatively stronger THG was found in vertical and planar spiral MoS_2_ as compared to 1L-MoS_2_	Ref [81]
WS_2_ spiral	SHG	1120–1330 nm	Direct CVD	SHG intensity quadratically increases with layer numbers	Ref [80]
WS_2_ spiral	THG	1120–1330 nm	Direct CVD	THG intensity increases with layer numbers. Power-dependent THG shows a slope of 3	Ref [80]
WS_2_ spiral	SHG	780–1330 nm	Direct CVD	SHG was strongly enhanced (∼100-fold increase) when the SHG resonated with the exciton states and when the excitation energy is slightly above the electronic bandgap	Ref [72]
Pyramid-like WS_2_ structure	SHG	810 nm	Direct CVD	Efficient edge SHG was recorded based on the enhanced light-matter interaction in whispering gallery mode cavities	Ref [77]
3D spiral WSe_2_	SHG	1064 nm	Direct APCVD	Enhanced SHG due to intense electric field confinement of surface plasmonic polaritons	Ref [75]
		CW-laser			
Few-layered MoSe_2_–WSe_2_	SFG/SHG	881 nm + 976 nm	ME and transfer	*χ* ^(2)^ is largest when the pump energy is around 1.4 eV which is close to the energy of interlayer excitons in this heterobilayer system	Ref [84]
		CW-laser			

CVD, chemical vapor deposition; APCVD, atmospheric pressure chemical vapor deposition; ME, mechanical exfoliation; FW, fundamental wavelength.

**Figure 2:**
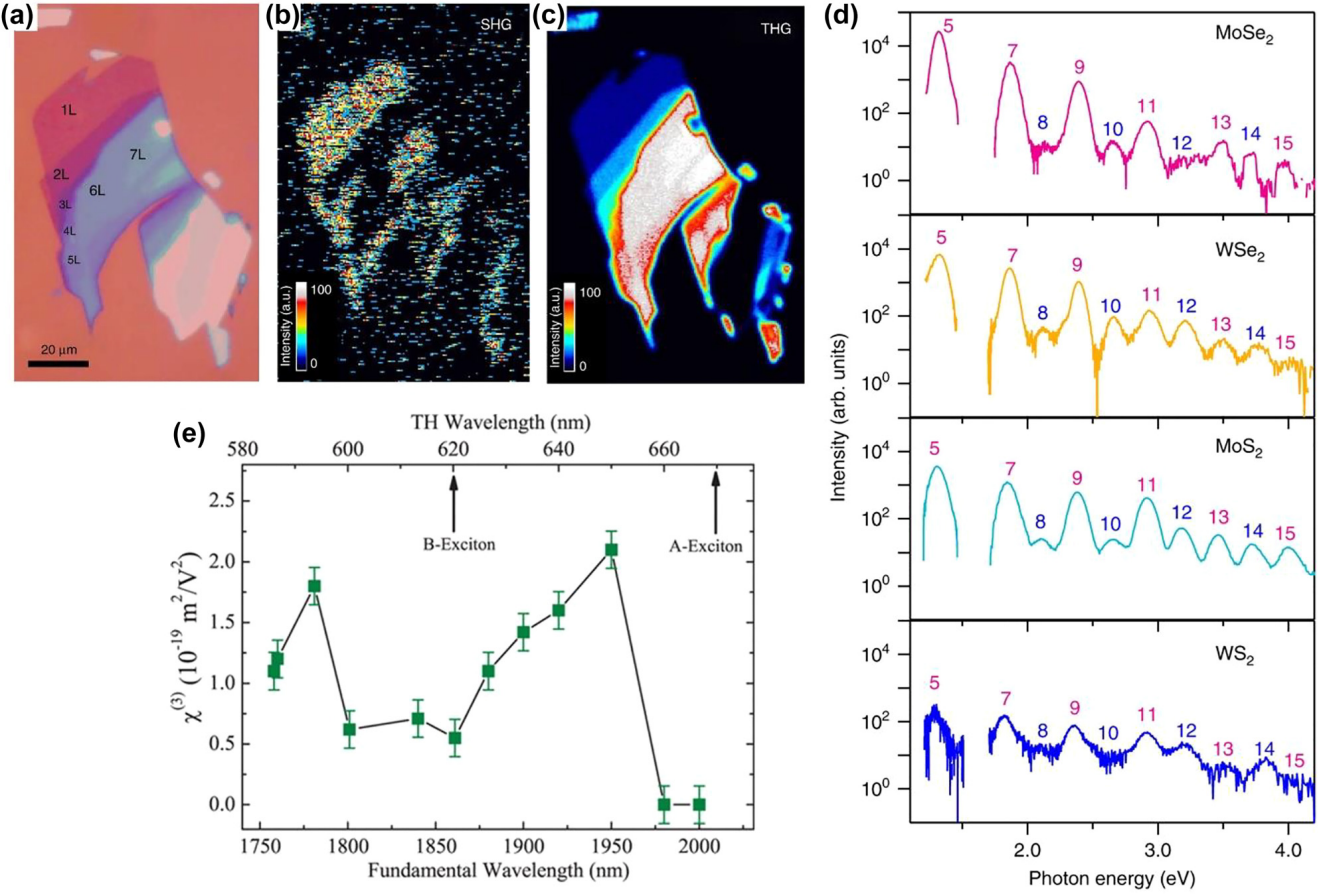
Representative studies of HG in TMDs. (a)–(c) Optical SHG and THG images of few-layer MoS_2,_ respectively, under the illumination of 1560 nm wavelength. (d) Experimentally measured HHG from the 1L-TMDs MoSe_2_, WSe_2_, MoS_2_, and WS_2_, respectively. The measurements were conducted at room temperature with mid-IR pulse excitation with a photon energy of 0.26 eV and a peak intensity of 1.7 TW cm^−2^. The polarization of the excitation laser beam was set parallel with the zigzag direction. The HG orders from the fifth to sixteenth order are labeled. (e) Dependence of *χ* ^(3)^ in 4.7 nm thick MoS_2_ on fundamental wavelength (bottom) and THG (top). The figures are reproduced with the permission: (a)–(c) ref. [156] © 2017 Nature Publishing Group (d) ref. [168] © 2019 Nature Publishing Group (e) ref. [157] © 2014 American Chemical Society.

In contrast to SHG, THG in TMDs can be observed in both odd-number and even-number structures. A significant advantage of the THG over the SHG is that the inversion symmetry does not need to be broken. Polarization resolved THG in TMDs is also different from SHG and does not depend upon the orientation of the 1L-TMDs belonging to the D_3h_ space group. The polarization of the recorded THG is usually the same as of the excitation polarization [7]. Wang et al. [157] in 2014 presented the first study of THG in ultrathin films of MoS_2_. Their measurement of the *χ* ^(3)^ as a function of light wavelength showed that excitonic resonances significantly enhanced the *χ* ^(3)^ (Figure 2e). In recent years, there have been increasing efforts on the THG in TMDs (Table 1). The *χ* ^(3)^ for 1L-TMDs is typically ∼ 10^−19^, which is three orders less than the value for graphene under the same measurement conditions [156, 157]. Despite this, most literature indicates that graphene also falls into the same range as MoS_2_ [158]. While it was mentioned previously that higher-order HG is weaker than lower-order HG, some studies [156] have demonstrated that the THG of MoS_2_ is larger than the SHG at a wavelength of 1560 nm. According to Säynätjoki et al. [156], this opposite behavior is due to the trigonal warping effect, which is a deviation from the purely isotropic band. It was also found [149] that the THG imaging technique can be efficient in resolving the grain boundaries in contrast to PL and Raman spectroscopy.

**Figure 3:**
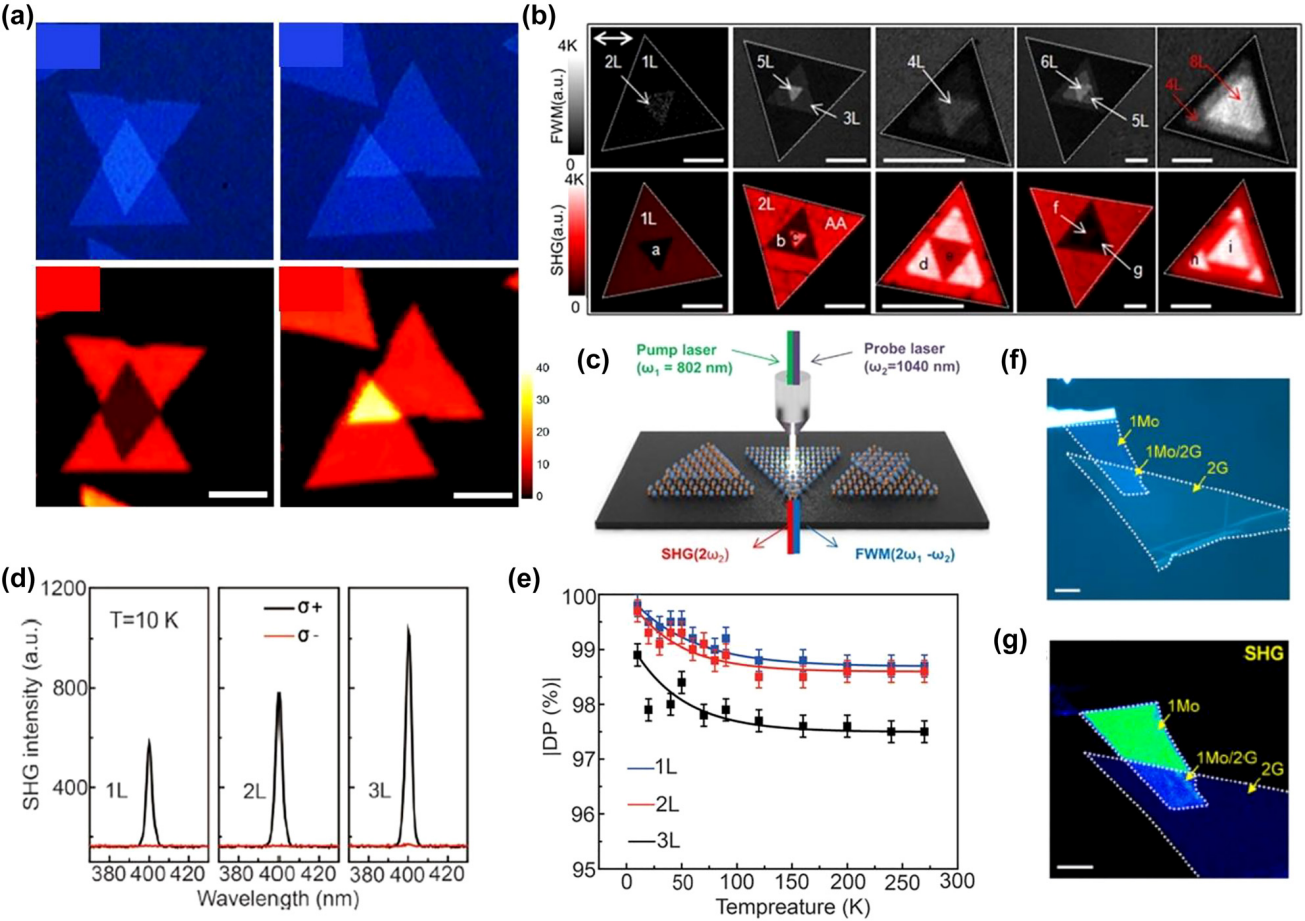
Representative works of HG in vdW heterostructures I. (a) Optical image of the stacked 2L-MoS_2_ in the first row for stacking angle 0° and 60° respectively with their SHG images in the lower row. The length of the scale bar is 5 µm. (b) Different types of MoS_2_ layer stackings resolved by collaborative four-wave mixing (FWM) and SHG imaging. (c) Schematic illustration of SHG and FWM after the interactions between the pump-probe laser beams with stacking-oriented MoS_2_ layers. (d) Circular polarization-resolved SHG spectra of the monolayer, bilayer (AA-stacked), and trilayer (AAA-stacked) WSe_2_ upon 800 nm excitation at temperature 10 K. (e) Degree of circular polarization (DP) of SHG as a function of temperature for monolayer, bilayer (AA-stacked), and trilayer (AAA-stacked) WSe_2_. (f) and (g) Optical and SHG images of graphene/MoS_2_ hetero bilayer, respectively. The figures are reproduced with the permission: (a) ref. [68] © 2014 American Chemical Society (b) and (c) ref. [74] © 2018 Nature Publishing Group (d) and (e) ref. [83] © 2020 Wiley-VCH Verlag GmbH & Co. KGaA, Weinheim (f) and (g) ref. [86] © 2016 American Chemical Society.

**Table 3:** Recent progress on enhancement or modulation of SHG in TMDs by the introduction of cavity and nanostructures.

TMD	Nanostructure	FW	Fabrication	SHG enhancement/modulation	Reference
1L-WS_2_	Au nanoholes metasurface	810 nm	CVD + transfer	2–3 orders enhancement	Ref [100]
1L-WS_2_	CdSe QDs	830 nm	ME + spin coated	Modulation	Ref [99]
1L-WS_2_	Dielectric MSs	804 nm	CVD + transfer MSs from suspension	20-Fold enhancement	Ref [103]
1L-WS_2_	Ag gratings	800 nm	APCVD + transfer	400 times enhancement	Ref [102]
1L-WS_2_	Optical fiber nanowire	1550 nm	CVD + transfer	20-Fold enhancement	Ref [112]
1L-WS_2_	Au nanoholes metasurface	1240 nm	CVD + transfer	10-Fold enhancement and steering SHG	Ref [111]
1L-WS_2_	Si metasurface	832 nm	ME + transfer	1140-fold enhancement due to BIC	Ref [115]
1L-WS_2_	Au metasurface	1210–1270 nm	CVD + transfer	Spatial control of SHG	Ref [110]
FL-MoS_2_	Au thin film	800 nm	ME + transfer	25 times enhancement	Ref [98]
1L-MoS_2_	DBR microcavity	818 nm	APCVD + transfer	10 times enhancement	Ref [87]
1L-MoS_2_	Core shell nanoparticles	810–1000 nm	CVD + transfer	∼1.88 times enhancement	Ref [105]
1L MoS_2_	TiO_2_ nanowire	800 nm	CVD + transfer	140 times enhancement	Ref [204]
1L MoS_2_	FP microcavity	680–1000 nm	CVD + transfer	3300 times enhancement	Ref [90]
1L MoS_2_	1D photonic crystals	794 nm	ME + transfer	170-fold enhancement	Ref [91]
1L-MoSe_2_	Si waveguide	1550 nm	ME + transfer	5 times enhancement	Ref [88]
1L-WSe_2_	Si PC cavity	1490 nm	ME + transfer	200-fold enhancement	Ref [92]
1L-WSe_2_	Au nanorod	700–800 nm	ME + transfer	Modulation	Ref [205]
1L-WSe_2_	Au trenches on flexible substrates	800 nm	CVD + transfer	7000-fold enhancement	Ref [93]
2L-WSe_2_	Au nanoparticle	800 nm	ME + transfer	Modulation & enhancement	Ref [97]
1L-MoS_2_	Si metasurfaces	850 nm	CVD + transfer	∼35-fold enhancement	Ref [202]

CVD, chemical vapor deposition; APCVD, atmospheric pressure chemical vapor deposition; ME, mechanical exfoliation; FW, fundamental wavelength; PC, photonic crystal; 1L, monolayer; 2L, bilayer; FL, few layer; FP, Fabry–Perot; DBR, distributed Bragg reflector; QDs, quantum dots.

**Table 4:** Different parameters to tune the HG in TMDs.

Engineering parameter	TMD with thickness	HG order	EW	Substrate	Fabrication	Enhancement/modulation	Reference
Exciton tuning	1L-WSe_2_	SHG	∼1490 nm	SiO_2_/Si	ME	4 times enhancement in *χ* ^(2)^	Ref. [67]
						15 times enhanced SHG (A-Exciton)	
	1L-WSe_2_		∼1416 nm	SiO_2_/Si	ME	Enhancement up to 3 orders at 4 K temperature (A-Exciton)	Ref. [207]
	1L-WSe_2_		∼1142 nm	SiO_2_/Si	ME	Enhancement up to 3 orders at 4 K temperature (B-Exciton)	Ref. [207]
	1L-MoS_2_		∼884 nm	Quartz	ME	∼8 times enhancement in *χ* ^(2)^ (C-Exciton)	Ref. [114]
	3L-MoS_2_		∼918 nm	Quartz	ME	∼6 times enhancement in *χ* ^(2)^ (C-Exciton)	Ref. [114]
	1L-MoSe_2_		1600 nm	SiO_2_/Si	PLD + selenization	∼5 times enhancement in *χ* ^(2)^ (A-Exciton)	Ref. [175]
	1L-MoS_2_		∼610 nm	SiO_2_/Si	CVD	∼4 times enhancement in *χ* ^(2)^ (B-Exciton)	Ref. [175]
	1L-MoS_2_		∼690 nm	SiO_2_/Si	CVD	∼4 times enhancement in *χ* ^(2)^ (A-Exciton)	Ref. [175]
	FL-MoS_2_	THG	595 nm	SiO_2_/Si	ME	Enhancement in *χ* ^(3)^ (near B-Exciton)	Ref. [157]
	FL-MoS_2_		650 nm	SiO_2_/Si	ME	Enhancement in *χ* ^(3)^ (near A-Exciton)	Ref. [157]
Electrical gating	2L-MoS_2_	SHG	∼995 nm	SiO_2_/Si	ME	60 times enhancement (−20–120 V)	Ref. [214]
	2L-MoS_2_		810 nm	SiO_2_/Si	ME	25 times enhancement (−12–15 V)	Ref. [241]
	1L-WSe_2_		∼1490 nm	SiO_2_/Si	ME	∼4 times enhancement (−80–80 V)	Ref. [67]
Strain	1L-MoSe_2_	SHG	820 nm	Acrylic	CVD	(−49% relative change in SHG per 1% uniaxial strain)	Ref. [224]
	1L-MoS_2_		800 nm	Flexible PEN	ME	Excellent modulation of SHG	Ref. [113]
	1L-TMD		895 nm	Flexible PEN	ME	Excellent modulation of SHG	Ref. [242]
	1L-TMD		800 nm	Flexible PEN	ME	Excellent modulation of SHG	Ref. [223]
	1L-WS_2_	THG	1288 nm	Acrylic	CVD	(−65% relative change in THG per 1% uniaxial strain)	Ref. [227]
Layer dependence	FL-2H-MoS_2_	SHG	1560 nm	SiO_2_/Si	ME	Negligible SHG at even layers and SHG decreases by increasing the odd number of layers from 1 to 5	Ref. [156]
	FL-2H-MoTe_2_	SHG	1550 nm	SiO_2_/Si	ME	Negligible SHG at even layers and SHG increases (odd: 1 to 5) and decreases (odd: 5 to 9)	Ref. [153]
	FL-2H-MoS_2_		810 nm	Fused SiO2	ME	Negligible SHG at even layers and SHG decreases by increasing the odd number of layers from 1 to 5	Ref. [62]
	FL-2H-MoS_2_		1370 nm	Quartz	ME	Negligible SHG at even layers and SHG stays same by increasing the odd number of layers from 1 to 5	Ref. [154]
	FL-3R-MoS_2_		1370 nm	Quartz	ME	The number of layers is directly proportional to the square of SHG magnitude	Ref. [154]
	FL-2H-MoS_2_	THG	1560 nm	SiO_2_/Si	ME	THG increases by increasing the number of layers from 1 to 7	Ref. [156]
	FL-2H-WS_2_	SHG	800 nm	SiO_2_/Si	ME	Negligible SHG at even layers and SHG decreases by an increasing odd number of layers from 1 to 7	Ref. [215]
	FL-2H-WSe_2_		800 nm	SiO_2_/Si	ME	Negligible SHG at even layers and SHG decreases by an increasing odd number of layers from 1 to 7	Ref. [215]
Valley tuning	1L-WSe_2_	SHG	1500 nm	SiO_2_/Si	ME	∼30 times enhancement (*σ*+ or *σ*− excitation)	Ref. [67]
	1L-WSe_2_		800 nm	SiO_2_/Si	CVD	∼3 times enhancement (*σ*− excitation)	Ref. [83]
	2L(AA)-WSe_2_		800 nm	SiO_2_/Si	CVD	∼8 times enhancement (*σ*− excitation)	Ref. [83]
	1L-WS_2_		800 nm	SiO_2_/Si	CVD	∼3 times enhancement (*σ*− excitation)	Ref. [83]
	2L(AA)-WS_2_		800 nm	SiO_2_/Si	CVD	∼7 times enhancement (*σ*− excitation)	Ref. [83]
	1L-MoS_2_		800 nm	SiO_2_/Si	CVD	∼2 times enhancement (σ- excitation)	Ref. [83]
	2L(AA)-MoS_2_		800 nm	SiO_2_/Si	CVD	∼4 times enhancement (*σ*− excitation)	Ref. [83]
	3L(AAA)-MoS_2_		800 nm	SiO_2_/Si	CVD	∼5 times enhancement (*σ*− excitation)	Ref. [83]

CVD, chemical vapor deposition; ME, mechanical exfoliation; EW, excitation wavelength; 1L, monolayer; 2L, bilayer; 3L, trilayer; FL, few layer; PEN, polyethylene naphthalate; PLD, pulse laser deposition.

Recent studies of HHG in solids have demonstrated that such nonperturbative nonlinearities can be achieved using a high-intensity laser excitation [159]. HHG in solids offers new opportunities to attain extreme ultraviolet sources and investigate the ultrafast electronic dynamics in the condensed phase [160], [161], [162], [163], [164], [165], [166]. HHG experiments in 2D materials commenced in 2017 with the investigation of 1L-MoS_2_ [167]. Liu and co-workers [167] excited the 1L-MoS_2_ on the fused SiO_2_ substrate with mid-IR pulses of 160 fs time at a photon energy of 0.30 eV well below the direct bandgap (1.8 eV). HG up to the 13th order was observed with an applied field intensity of 2.2 TWcm^−2^. This enhanced HHG efficiency in the 1L-MoS_2_ was attributed to the strong electron and hole Coulomb interactions. In another work, HHG efficiency up to the 18th order was achieved by Yoshikawa et al. [168]. It was suggested that resonant enhancement of HHG with the interband optical transition in their experiment was due to the band nesting effects. Furthermore, the symmetry analysis revealed that valley polarization and anisotropic band structure result in HHG polarization [168]. Such a high HHG in 1L-TMDs indicates the potential to examine attosecond and strong-field phenomena in materials of reduced dimensionality. It may also result in the externally controlled HHG using electric gating, layer stacking, twisting, strain, etc., in 1L-TMDs.

## 4 HG in TMDs heterostructures

### 4.1 HG in twisted and vdW heterostructure TMDs

Various TMDs heterostructures have been designed by assembling the individual single layers into multilayer structures with atomically sharp interfaces, without interdiffusion of atoms, lattice parameter constraints, and control over the layered components [179, 180]. In TMD heterostructures, constituent TMDs layers influence each other’s properties through the proximity effect, and interlayer physics produces new phenomena such as interlayer excitons [138, 181, 182]. In addition, these structures provide a means of controlling the electronic properties of the materials. For instance, when 1L-WS_2_ or 1L-WSe_2_ are stacked with 1L-MoS_2_, the bandgap changes from direct to indirect. Twisting in homo- and hetero-2L-TMDs structure provides a new degree of freedom to control their physical properties, resulting in a wide variety of novel phenomena such as unconventional superconductivity [183], non-trivial topological Chern number [184], and Moiré excitons [185, 186], etc. Moreover, the interlayer twisting in 2D vdW materials determines the crystal space groups. It opens completely new dimensions to engineer the symmetry, especially when the inversion symmetry is broken with a relative twist angle other than 2*mπ*/*N*, where *N* is the *N*-fold rotational symmetry and m is an integer [187].

**Figure 4:**
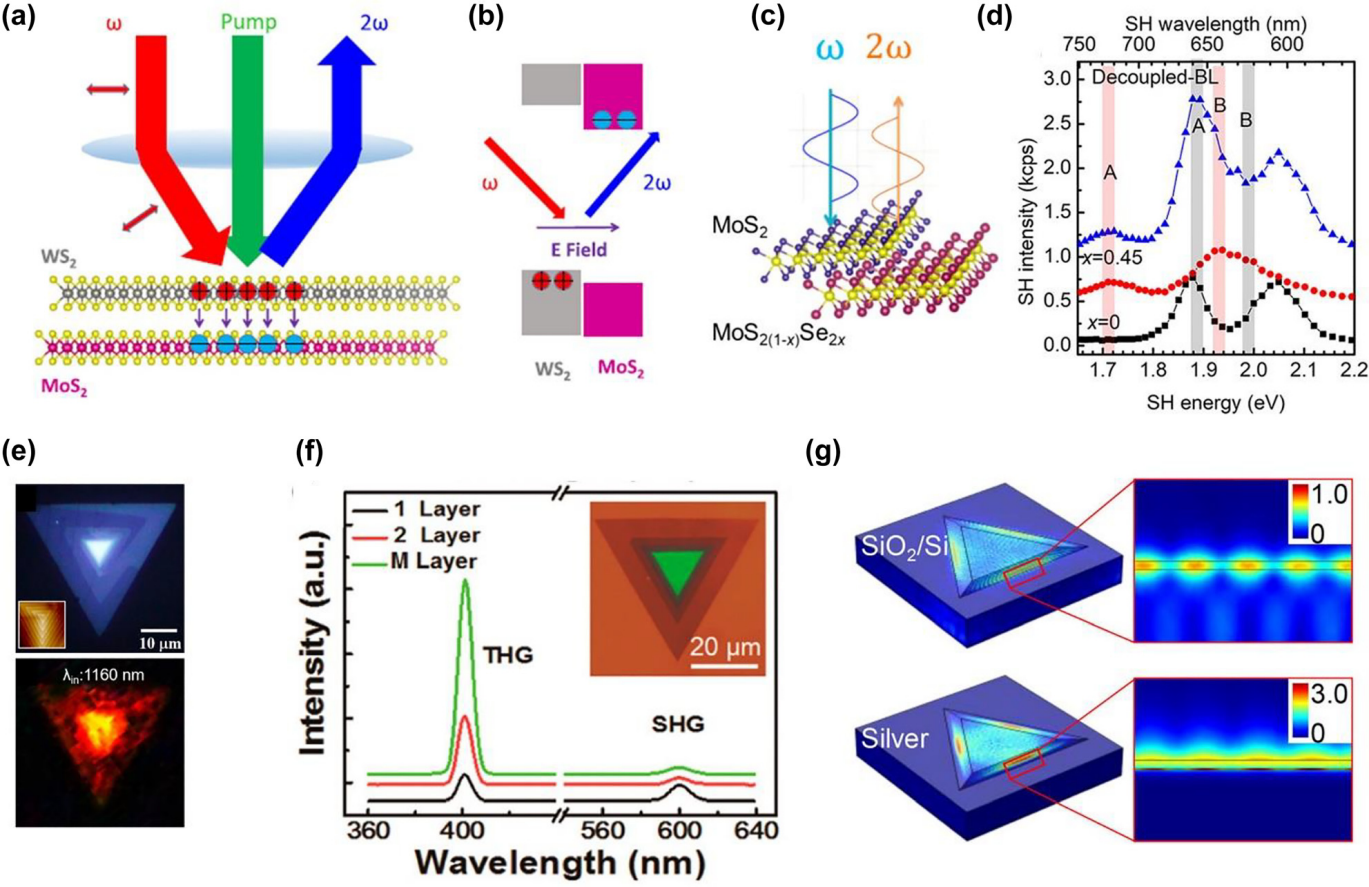
Representative works of HG in vdW heterostructures II. (a) Field-induced SHG when a pump pulse impinges on WS_2_/MoS_2_ heterostructure and produces electrons and holes. The separation of the charge carriers generates an electric field that is sensed by SHG. (b) Band alignment of WS_2_/MoS_2_ and expected charge separation. (c) Generation of SHG in MoS_2_/MoS_2(1−*x*)_Se_2*x*_ heterostructure. (d) Wavelength dependent SHG spectra for 1L-MoS_2_ in black, 1L-MoS_2(0.55)_Se_(0.45)_ decoupled hetero 2L-MoS_2_/MoS_2(1−*x*)_Se_2*x*_ (intercalated with a 10-nm-thick SiO_2_ layer) in blue color. (e) Optical image of the spiral WS_2_ flake on the quartz substrate (top); inset is the AFM image of the spiral WS_2_ flake. SHG image at 1160 nm excitation wavelength of fs-laser for the corresponding top optical image (bottom). (f) Dependence of SHG and THG in WS_2_ on the number of layers. Inset: the optical image of spiral WS_2_ stacking structure in which the top layer is green. (g) Calculated electric field distributions in a pyramid-like multilayer WS_2_ on SiO_2_/Si (up) and Ag (bottom) substrates, respectively. The figures are reproduced with the permission: (a) and (b) ref. [73] © 2019 AIP Publishing (c) and (d) ref. [76] © 2020 American Chemical Society (e) ref. [72] © 2020 American Chemical Society (f) ref. [80] © 2017 American Chemical Society (g) ref. [77] © 2018 American Chemical Society.

The study of the HG in TMDs heterostructures (Figures 3 and 4) was started in 2014 (Table 2). Hsu et al. [68] investigated SHG from CVD-grown homo- and hetero-2L-TMD with an arbitrary stacking angle. It was found that the SHG from the twisted bilayers is a coherent superposition of the SHG fields from the constituent layers with a phase difference that depends on the stacking angle. Their experiment also showed that an interference effect of this kind is insensitive to the constituent layers of materials. In another work [82], experimental and theoretical evidence that interlayer twists can be used to tune indirect and direct band gaps in 2L-MoS_2_ was provided. Also, it was shown that the SHG in 2L-MoS_2_ can be efficiently tuned by changing the interlayer twisting angle. Li et al. [86] combined the SHG with sum–frequency difference (SFG) and four-wave mixing (FWM) to study the nonlinear optical response of the MoS_2_-graphene heterostructures (Figure 3f). It was found that all three kinds of nonlinear processes were sensitive to the number of layers, crystallinity, and interlayer coupling. The graphene-MoS_2_ heterostructures indicated a strong quenching of SHG-SFG, revealing a strong interlayer coupling between MoS_2_ and graphene. Lin and co-workers [77] recorded a significant enhancement of SHG in pyramid-like multilayer WS_2_.

Enhancement of SHG was accomplished by taking advantage of the enhanced light–matter interaction generated by a highly confined field within the whispering gallery mode. An extraordinary edge SHG was produced in pyramid-like multilayer WS_2_ due to partial destructive interference of nonlinear polarization between the neighboring atomic layers. Increasing the number of atomic layers leads to a maximum light–matter interaction and an enhanced SHG by 40 times compared to the SHG from 1L-WS_2_. According to the study, hybridizing the WGM mode with plasmonics can increase SHG over 800 times [77]. Researchers have also examined spiral TMD structures for the manipulation of HG. According to Fan et al. [80], CVD-grown spiral structures of WS_2_ exhibit strong SHG and THG. It was found that SHG intensity increases quadratically with layer number. Similarly, in another work [78], it is shown that vertical and planar spiral MoS_2_ nanosheets exhibit stronger SHG and THG than the 1L-MoS_2_. A strong SHG was achieved in hybrid 3D spiral WSe_2_ plasmonic structures [75] due to strong electric field confinement. In 3D spiral WSe_2_ nanostructures, the constructive interference of SHG fields between adjacent atomic layers in conjunction with enhanced light–matter interaction results in extremely high conversion efficiency of SHG fields. SHG is found to undergo a two-order enhancement in spiral WS_2_ structure near the ultraviolet region in a recent wavelength and temperature-dependent investigation [72]. In addition to C and D exciton resonances, the reason for this high SHG intensity was attributed to the resonance of large interband Berry connections, leading to certain transitions within the high energy spectral regime.

Yao et al. [73] in 2019 suggested that SHG can directly probe electric fields induced by the charge transfer. The electric field generation was explained by separating electrons and holes in the two 1L-MoS_2_/WS_2_ heterostructures, leading to the SHG (Figure 4a). The measurement results agreed with the dynamics revealed by transient absorption. Another research group investigated the effect of interlayer coupling and band offset on SHG in 3R homo bilayer (MoS_2_/MoS_2_) and hetero-bilayer (MoS_2_/MoS_2(1−*x*)_Se_2*x*_) [76]. Due to the band structure modification that occurs through the coupling between interlayers, the measured results reveal that the SHG response from the coupled homo bilayer cannot simply be explained by superimposing SHG fields from constituent layers. The band offset creation in their heterostructure geometry indicated that the overall SHG intensity is strongly dependent on the phase mismatch between the SHG fields from MoS_2_/MoS_2(1−*x*)_Se_2*x*_. Another exciting report [84] investigated continuous wave-induced SHG and SFG from 1L-TMDs and their heterostructure using pump fluences considerably lower than those used in conventional pulsed laser-induced SHG experiments. Circular polarization-dependent investigation [83] was performed on the different types of monolayer and heterostructure TMDs (as-grown MoS_2_, WS_2_, WSe_2_ layers, spirals, and WS_2_/MoS_2_ heterostructures). Due to the inversion symmetry breaking, a high degree of SHG polarization was observed in 3R TMDs few-layers and spiral structures at room temperature. According to the study, the interlayer coupling does not affect the valley-dependent SHG at room temperature. However, it increases at low temperatures due to the suppression of interlayer scattering. Due to exceptional sensitivity to the inversion symmetry, researchers utilized SHG as a characterization tool to find the symmetry and twisted angle between the lateral and the vertical vdW heterostructures by direct imaging [74, 79].

### 4.2 Enhancement of HG in TMDs by nanostructures

#### 4.2.1 Cavity

The weak interaction with light limits the nonlinearity of the TMDs-based devices due to the atomically thin nature of these materials. Hence high-power lasers are needed to trigger the nonlinearity in these devices. Consequently, improving the nonlinear efficiency in TMDs for real-world applications and reducing the threshold power of pumping lasers are of great importance. Plasmonic and photonic cavities and resonators have been used to enhance the light–matter interaction in TMDs through the spatial and temporal confinement of electromagnetic fields. Such resonators are characterized by the quality factor (*Q*) and mode volume (*V*) [69]. The quality factor roughly corresponds to the mean number of round-trips of a photon in the resonator. For a large *Q*-factor, even a tiny quantum system like the atomically thin TMDs acquires a considerable probability of interaction with the cavity photon. In its turn, the smaller mode volume gives rise to the increased electric field of the mode per photon, increasing the light–matter interaction effects such as HG.

**Figure 5:**
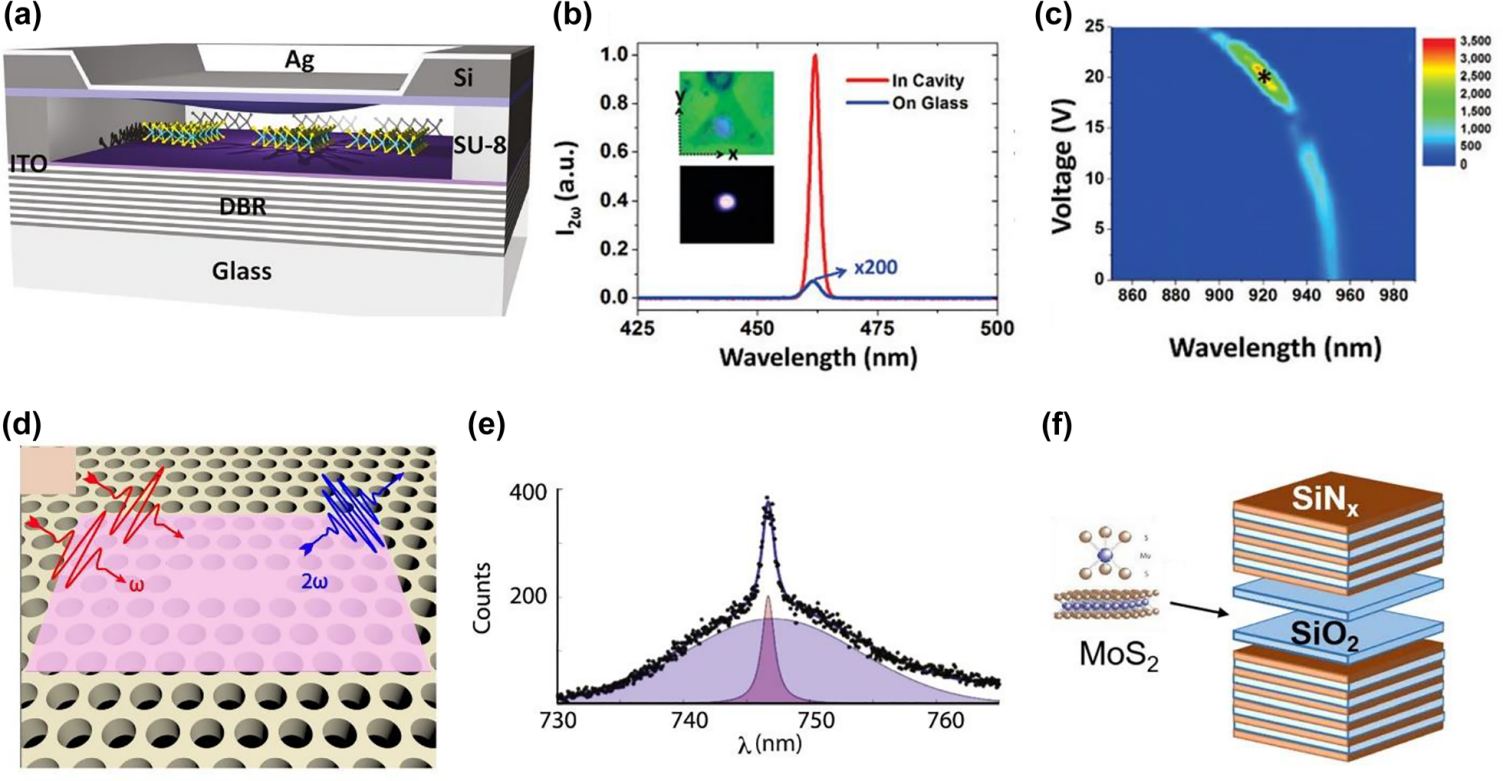
Cavity-enhanced HG in TMDs. (a) Schematic of the doubly resonant cavity structure made by the dielectric mirror on a glass substrate and a Ag mirror on a suspended nitride membrane. (b) SHG generated from the 1L-MoS_2_ inside the cavity (solid red line) and SHG from the 1L-MoS_2_ on a glass substrate (solid blue line). The pump wavelength used in this measurement was 925 nm, and the tuning voltage was set as 20 V. The insets in the graph show the spot of the SHG from the 1L-MoS_2_ in the cavity under bright (top) and dark (bottom) fields. (c) The total output SHG power enhancement factor SHG from cavity region compared to MoS_2_ on a glass substrate as a function of voltage and wavelength. (d) The schematic illustration SHG generation from Si photonic crystal cavity coupled to the 1L-MoSe_2_©. (e) SHG spectra of MoSe_2_ loaded Si photonic crystal cavity indicating the Gaussian background with a Lorentzian fit specifying the cavity resonance. (f) Schematic diagram of microcavity with distributed Bragg reflectors (DBR) mirrors consisting of interleaving SiN_x_ and SiO_2_ and SiO_2_ layers. The figures are reproduced with the permission: (a)–(c) ref. [90] © 2016 American Chemical Society (d) and (e) ref. [92] © 2016 IOP Publishing Ltd (f) ref. [87] © 2016 Optical Society of America (OSA).

In recent years, the emphasis has been placed on improving the SHG from 1L-TMDs coupled to single cavity modes (Table 3). Fryett et al. [92] observed an enhanced SHG by inserting the 1L-WSe_2_ on the Si photonic crystal cavity, Figure 5d. The cavity was designed so that its cavity mode appeared at 1490 nm. A strong SHG was observed around 745 nm wavelength (Figure 5e) when the cavity + WSe_2_ was resonantly excited by a pulsed laser. The measured SHG enhancement was ∼200 compared to SHG of 1L-WSe_2_ on Si. A primary reason for the low enhancement was the lack of a low Q-factor of the cavity and the absence of a cavity mode at the second harmonic frequency. Additionally, the Si absorbs a considerable amount of SHG signals.

In another investigation, Day et al. [87] integrated the 1L-MoS_2_ inside the distributed Bragg reflector (DBR) cavity (Figure 5f) and enhanced SHG of the MoS_2_ up to 10 folds. The minor enhancement was due to the large cavity mode volume and low *Q*-factor. Designing a doubly resonant cavity can alleviate the problem of enhancement. Developing such a mode structure is not easy because inevitable fabrication errors detune the cavity from the operational wavelength. Yi et al. [90] fabricated a mechanically tunable Fabry–Perot cavity whose top mirror was a capacitively actuated Ag mirror, whereas the lower mirror was a DBR cavity, Figure 5a. The purpose of capacitive tuning was to alter the cavity length and, therefore, cavity resonances. According to ref. [90], mechanical tuning can match both the fundamental and second harmonic modes to the desired wavelengths. Despite the low cavity *Q*-factor, SHG was enhanced up to ∼3300 times. Furthermore, it was predicted that further improvement in the structure could be achieved by reducing the mode volume and improving the cavity *Q*-factor.

#### 4.2.2 Plasmonic and dielectric nanostructures

**Figure 6:**
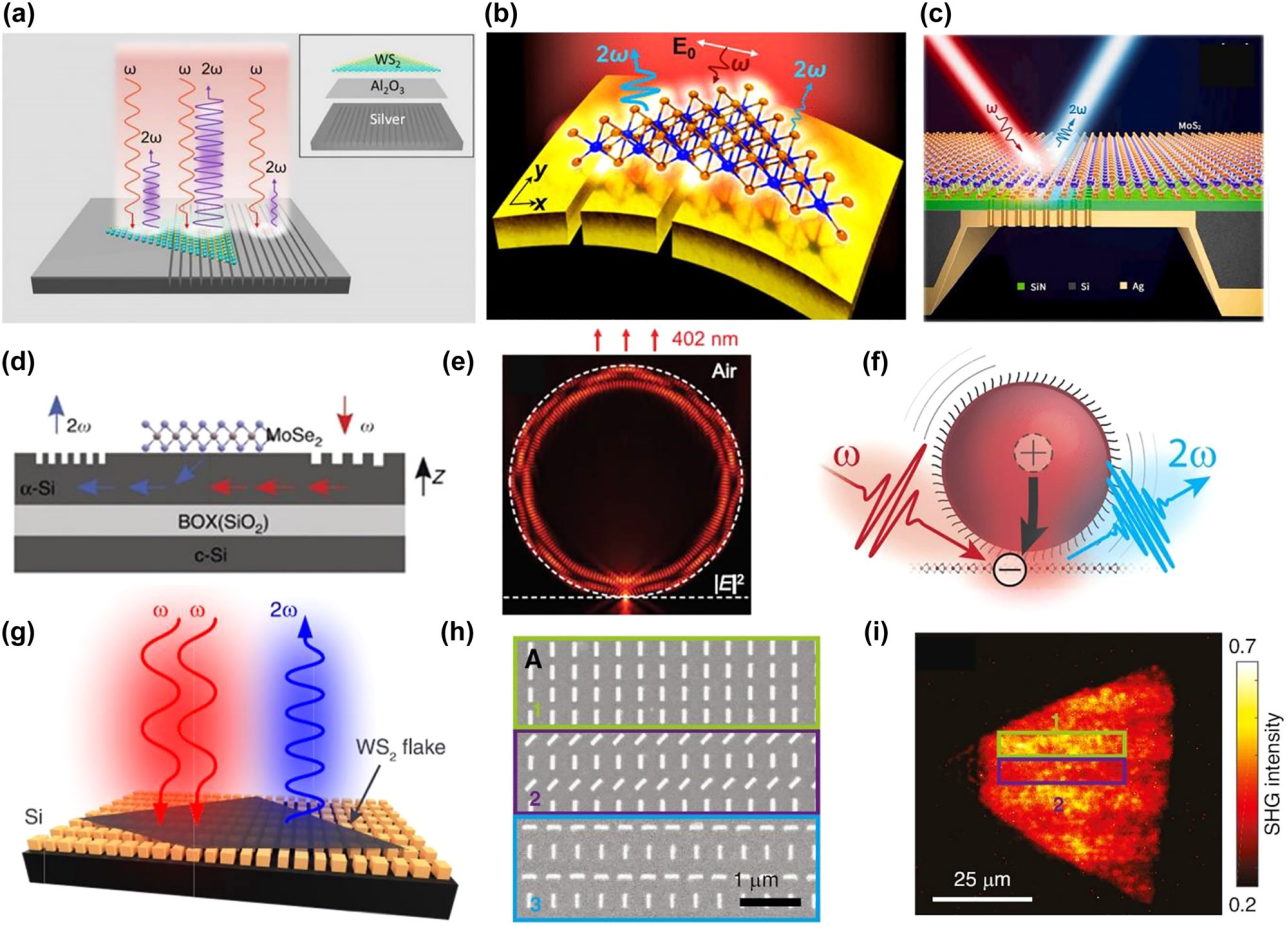
Nanostructure enhanced HG in TMDs. (a) WS_2_–Ag metasurface. It shows that a weak SHG was produced when a Ag grating structure was present; however, a stronger SHG is generated when you coupled WS_2_ with a Ag grating structure. The inset represents a schematic of a layered hybrid structure. (b) Strong SHG emitted from 1L-WSe_2_ when it was coupled to the Au trenches on the flexible substrate. (c) Generation of SHG 1L-MoS_2_ on suspended metallic nanostructures by plasmonic resonances. (d) MoSe_2_ integrated Si waveguide hyb© structure. (e) Simulated field distributions of the SHG. (f) Modulation of the SHG in CdSe quantum dots coupled to 1L-WS_2_. (g) SHG generation from 1L-WS_2_ on the Si metasurface. The hybrid structure hosts BIC to enhance the SHG in 1L-WS_2_. (h) and (i) Scanning electron microscopic (SEM) image of the Au nanorods orient in the different direction marked by 1 (green), 2 (purple), and 3 (blue) rectangles. The corresponding SHG spatial map of regions 1 and 2, indicated by green and purple rectangle when 1L-WS_2_ was coupled to the Au nanorod metasurface. The figures are reproduced with the permission: (a) ref. [102] © 2018 Wiley-VCH Verlag GmbH & Co. KGaA, Weinheim (b) ref. [93] © 2018 American Chemical Society (c) ref. [89] © 2021 De Gruyter (d) ref. [88] © 2017 Nature Publishing Group (e) ref. [103] © 2018 WILEY-VCh Verlag GmbH & Co. KGaA, Weinheim (f) ref. [99] © 2018 American Chemical Society (g) ref. [115] © 2020 American Chemical Society (h) and (i) ref. [110] © 2019 De Gruyter.

Many researchers have used plasmonic nanostructures to enhance the interaction between light and matter in 1L-TMDs since their electromagnetic fields can be localized and enhanced into a nanoscale region (near field region) [188], [189], [190], [191]. Shi et al. [102] improved the SHG in 1L-WS_2_ by incorporating it on the Ag nanogrooves with subwavelength pitch, Figure 6a. A 400-fold boost in SHG was achieved with a conversion efficiency of ∼2 × 10^−5^. This effect was observed when the surface plasmon mode and SHG frequency were in resonance with the C exciton in WS_2_. Furthermore, another study [93] reported that a giant SHG could be obtained in WSe_2_ by coupling it to Au film with trenches supporting lateral gap plasmon resonances at ∼800 nm. The proposed hybrid system was quite flexible, and it provided an increase of ∼7000 folds in the SHG without broadening the SHG peak at room temperature. Similarly, SHG was also enhanced in 1L-MoS_2_ by coupling it to the core (Au) shell (SiO_2_) monomer and dimers [105]. Furthermore, the plasmonic metasurfaces are used to modulate the valley-selective SHG from the 1L-TMDs spatially. For example, Spreyer et al. [110] investigated the valley-exciton locked SHG from the TMDs based hybrid structure (1L-WS_2_ + Au metasurface), and coherent steering of SHG was achieved in WS_2_. Chen and co-workers [100] enhanced SHG by coupling a Au nanohole metasurface with a 1L-WS_2_. This hybrid system achieved a second-order susceptibility equal to 10^−10^ mV^−1^, ∼2–3 orders larger than typical plasmonic metasurfaces. In this work, a series of nonlinear metalenses with focal lengths of 30 μm, 50 μm, and 100 μm was also demonstrated experimentally, providing a solid proof for generating and manipulating SHG based on these TMDs based hybrid metasurfaces. Similar plasmonic enhancement and manipulation of SHG from 1L-TMDs were realized by several other groups [97, 192].

Dielectric nanostructures have been introduced as an alternative to the plasmonic nanostructures because they offer almost dissipative losses in the visible and IR regimes and can support both electric and magnetic resonances [13, 56, 193], [194], [195], [196], [197], [198], [199]. In 2017, Chen et al. [88] incorporated 1L-MoSe_2_ onto the Si waveguide, and an increased length of nonlinear interaction with TMDs was achieved with a phase-matched SHG. The observed enhancement in the SHG from the MoSe_2_ loaded Si waveguide was 5-fold when 1L-MoSe_2_ was excited by the evanescent waveguide mode compared to excitation from free space. In another experiment [115], using a resonant Si metasurface supporting the bound state in continuum (BIC) effect, the effective *χ* ^(2)^ of the 1L-WS_2_ was increased. BIC is a simple approach to engineer the radiative losses of various all-dielectric nanostructures [200, 201]. The enhanced SHG of up to three orders of magnitude compared to the SHG from 1L-WS_2_ on top of a flat Si film of the same thickness was demonstrated. Dielectric microspheres were also employed to enhance the SHG from 1L-WS_2_ [103]. SHG enhancement (20 times) in the WS_2_ loaded SiO_2_ microsphere was attributed to their increased field intensity and enlarged collection efficiency. Similarly, Chen and co-workers [112] integrate 1L-WS_2_ to an optical fiber nanowire for broadband enhancement of light–matter interactions through the evanescent field coupling effects in the optical fiber nanowire. An enhanced SHG by 20 times was found in WS_2_ loaded optical fiber nanowire compared to the bare optical fiber nanowire. Quantum dots [99] were also employed to modulate the SHG from 1L-WS_2_. In addition, several other structures like cellulose nanofibrils [99], small chitosan [108], Si metasurfaces [202], and TMDs nanostructures [203] were employed to manipulate or enhance the HG in TMDs (Table 3).

## 5 Controlling the HG in TMDs

### 5.1 Excitonic tuning of SHG

An exciton is a bound state of positive (holes) and negative (electrons) charges [51]. They are usually formed when light interacts with semiconductors. In conventional bulk semiconductors like Si, the large dielectric screening and small quasiparticle effective mass lead to the small exciton binding energies (1–10 meV). However, unlike conventional semiconductors, excitons exhibit binding energies equal to a few hundred of meV due to efficient Coulomb interaction in atomically thin dimensions and reduced dielectric screening [206]. In addition, they host valley polarization, and their atomically thin nature allows them to be tuned by several external stimuli, thus enabling their potential use in 2D photonic devices [58]. TMDs have nonlinear optical properties that are influenced by excitons in the same manner as their linear optical properties.

Many investigations focused on excitonically modulated or enhanced SHG and THG in recent years have been reported. In 2013, Malard et al. [114] demonstrated that SHG can be enhanced up to 8 and 6 times for the 1L and 3L-MoS_2_, respectively, when SHG energy was in resonance with C-exciton. Le et al. [175] measured five times enhancement in 1L-MoSe_2_ at A exciton resonance compared to the SHG at B-exciton resonance. They also measured the exciton resonance dependent SHG in MoS_2_ and found that the magnitudes were similar to the A and B exciton SHG, in contrast to the excitonic dependent SHG observed in MoSe_2_. In low temperature (4 K) exciton-dependent SHG spectra for 1L-MoS_2_, it was found [207] that the SHG enhancement was ∼3 times at exciton A and B. This enhancement was attributed to the unusual combination of an electric dipole and magnetic dipole transitions. The excitonic SHG in 1L-TMDs can also be actively tuned by elemental doping. In 2017, Le et al. [151] found that the SHG of MoS_2(1−*x*)_Se_2*x*_ at the excitonic resonance can be tuned efficiently by changing the doping concentration of selenium (Se). As can be seen in the case of the MoS_2_, the SHG is strong only at A exciton resonance, whereas the doping concentration of Se changes, the SHG at B exciton rises and almost becomes equal to the SHG at A when *x* = 0.30 in MoS_2(1−*x*)_Se_2*x*_. The peak of SHG at A exciton almost disappeared at *x* = 62. This study demonstrated that alloying MoS_2_ with Se considerably enhances and broadens the effectiveness of SHG. Alternatively, the exciton tuning can also be carried out by electron doping. Seyler et al. [67] described that SHG can be actively controlled in the 1L-WSe_2_ field-effect transistor. The strength and frequencies of the resonance SHG in 1L-WSe_2_ were tuned by controlling the electrostatic doping. Similarly, several other experimental studies [148, 169, 172, 208] have been conducted to modulate or enhance SHG in 1L-TMDs by excitons.

**Figure 7:**
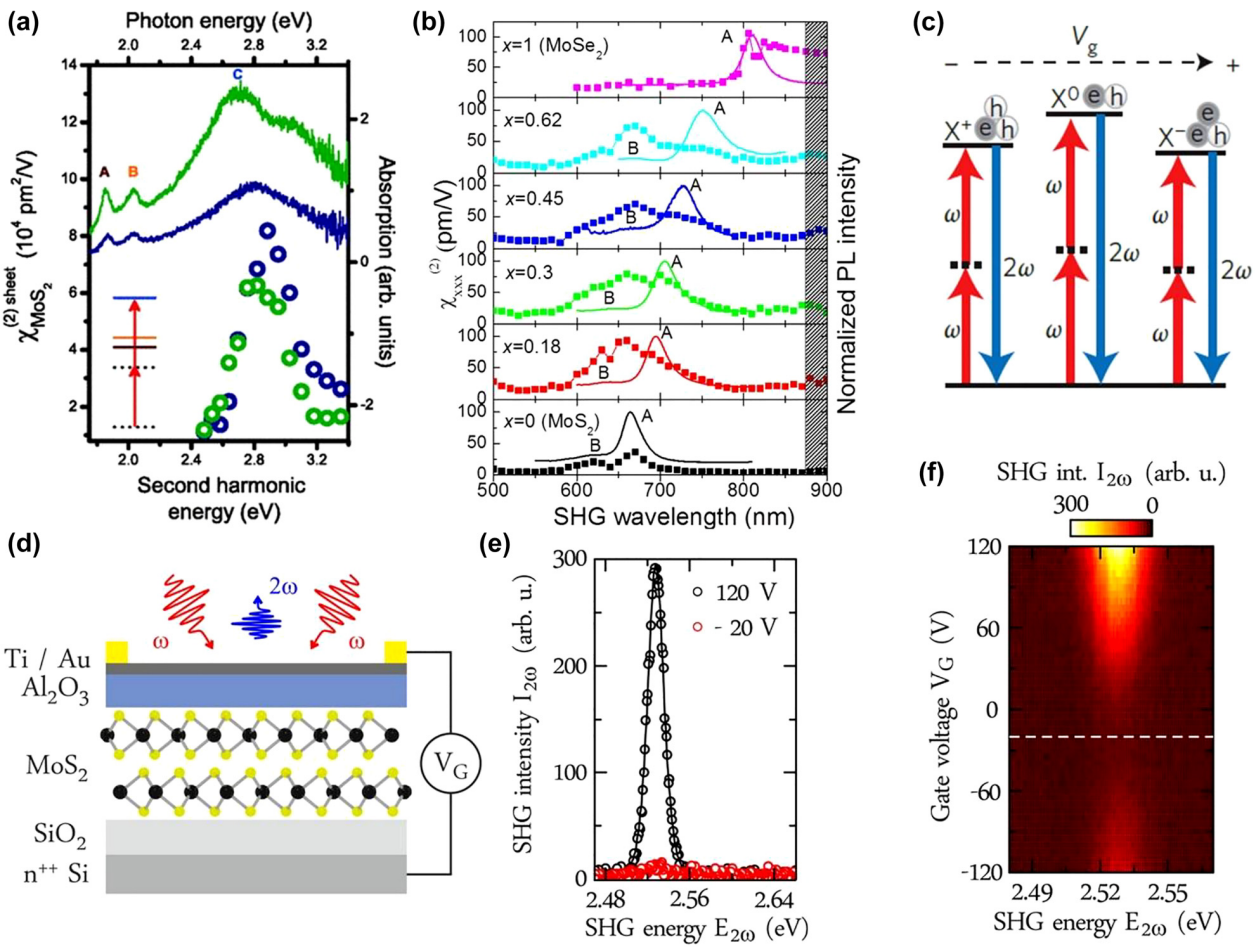
Excitonic and gate tuning of HG in TMDs. (a) Graph illustrating the *χ* ^(2)^ of MoS_2_ as a function of pump laser energy for single layers (blue circles) and trilayers (green circles), together with the measured linear absorption shown on the right. The solid blue line represents the linear absorption spectrum of 1L-MoS_2,_ while the solid green line indicates the absorption spectrum of 3L-MoS_2_. In the inset, you can see how the two-photon energy resonates with the C absorption peak in 1L-MoS_2_. (b) *χ* ^(2)^ of MoS_2(1−*x*)_Se_2*x*_ alloy as a function of SHG wavelength with PL spectra on the right side of the graph for different compositions of Se. The dotted line indicates the *χ* ^(2)^ spectra, while the solid line indicates PL spectra. (c) Illustration of gate-dependent excitonic and trionic enhancement of SHG. (d) 2L-MoS_2_ micro-capacitor shown schematically. (e) SHG spectra showing the maximum magnitude at VG (gate voltage) = 120 V and minimum VG = −20 V, indicating a clear voltage dependence of the SHG intensity. Inversion symmetry of the 2L-MoS_2_ is maximally broken for VG = ±120 V, whereas it is restored for VG = −20 V. (f) Graph illustrating the SHG intensity as a function of the applied gate voltage and the SHG energy for a 2L-MoS_2_ system excited at *E*_ω_ = 1.246 eV. At VG = −20 V, the dashed white line indicates the gate voltage at which minimum SHG can be observed. The figures are reproduced with the permission: (a) ref. [114] © 2013 American Physical Society (b) ref. [151] © 2016 American Chemical Society (c) ref. [67] © 2015 Nature Publishing Group (d)–(f) ref. [214] © 2016 American Chemical Society.

### 5.2 Gate tuning of SHG

Bulk semiconductors are unsuitable for optical modulators since their refractive index constants vary by only 0.01% with gate voltage [209, 210]. On the contrary, atomically thin semiconductors such as 1L-TMDs offer an excellent electrically tunable optical response. For instance, the PL intensity can be well-tuned with gate voltage, indicating 1L-TMD, a potential candidate for the electro-optical modulator [211, 212]. Similarly, it is expected that gate tuning can also alter the nonlinear optical response of the 1L-TMDs. To accomplish this objective, researchers investigated the SHG of 1L-TMDs as a function of gate voltage. In 2015, Seyler et al. [67] reported that the SHG intensity of 1L-WSe_2_ at A exciton resonance can be tuned up to four times and over one order at room and low temperature, respectively. Such tunability was attributed to the strong exciton charging effects in 1L-semiconductors which offer exceptional control over the oscillator strengths at the exciton and trion resonances. The gate voltage is also utilized to break the inversion symmetry in the 2H 2L-WSe_2_ based Back-gated field-effect transistors [213]. Under identical measurement conditions, the SHG from 2L-WSe_2_ was 1000 times lower than that from 1L-WSe_2_ at gate voltage equal to −40 V. The authors identify the generation of SHG as charge-induced SHG, which differs from electric field-induced SHG. Due to the asymmetric behavior of WSe_2_, SHG was generated and enhanced as a result of the presence of mobile charges on the d-shells of W. This exceptional property is only possible in TMDs. Similarly, Klein et al. [214] have also demonstrated that inversion symmetry is broken in 2H 2L-MoS_2_ with gate voltage (Figure 7d). The electric field-induced SHG in 2H 2L-MoS_2_ was reported. It was stated that hybridization is necessary between the two individual layers for generating the electric field-induced SHG in 2L-MoS_2_. An enhanced SHG by 60 times was produced at *E*_2*ω*_ = 2.49 eV. Although SHG gate tuning is well known in TMDs, these works demonstrate that a deeper understanding of exciton charging effects still needs to be conducted.

### 5.3 Thickness and phase dependent HG

**Figure 8:**
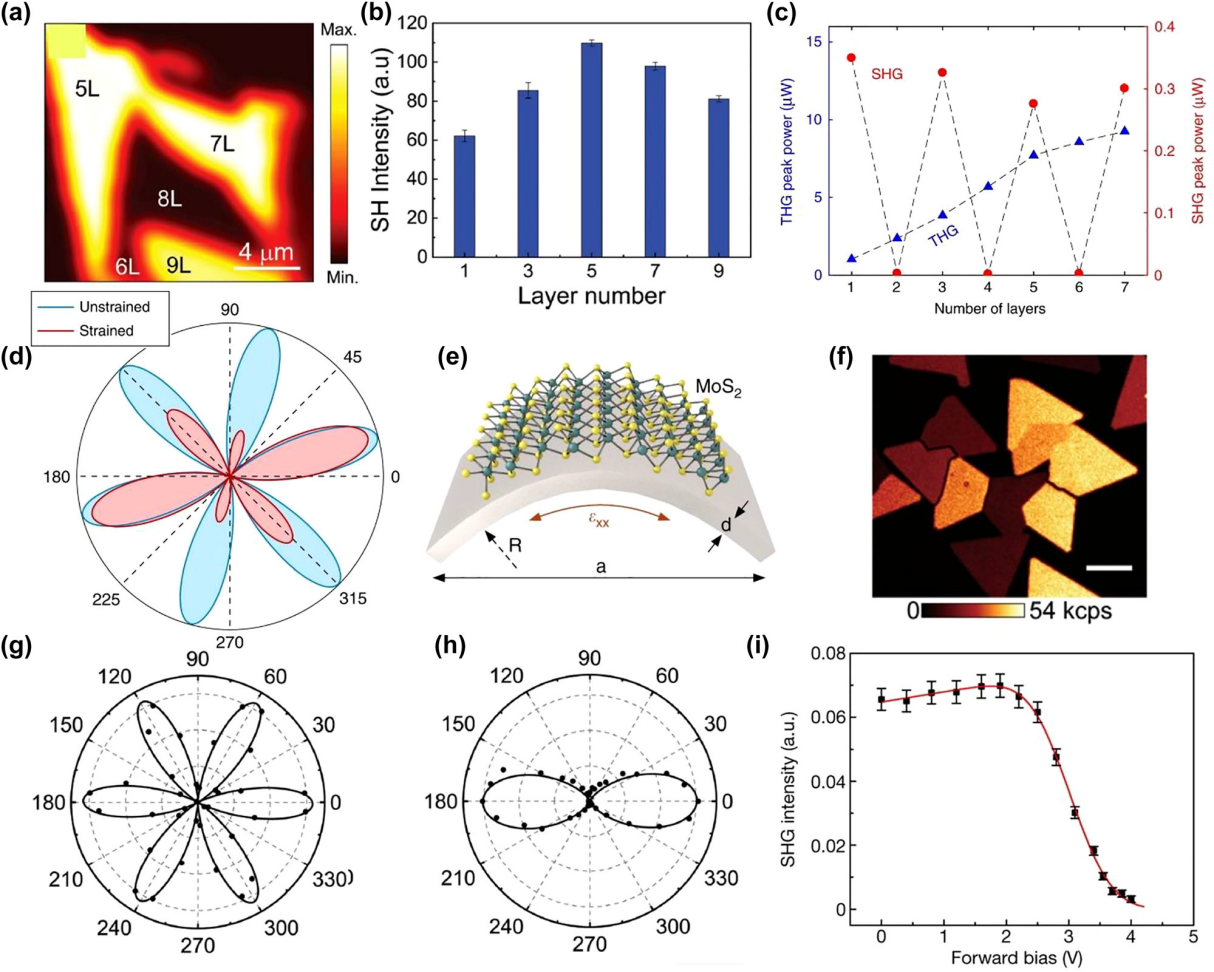
Layer, phase, and mechanical tuning of HG in TMDs. (a) SHG spatial map of MoTe_2_ flakes with different thicknesses. (b) An illustration of the layer-dependent SHG of MoTe_2_ with error bars denoting the uncertainty. (c) Layer-dependent SHG and THG spectra for MoS_2_ flakes. (d) Polarization resolved SHG spectra for unstrained and strained (1% tensile strain) in TMD crystal. (e) The two-point bending method is illustrated schematically. (f) SHG spatial map of the CVD grown MoS_2_ indicating a clear image of edge states. The length of the scale bar is 10 µm. (g) and (h) Polarization resolved SHG spectra of few-layer MoTe_2_ before and after the laser irradiation. (i) SHG intensity for 1L-MoTe_2_ under a forward bias varying with the gate voltage. Increasing the voltage bias from 2 to 4 V results in a significant reduction in the SHG intensity. This decline can be attributed primarily to the formation of the 1T′ phase, which maintains inversion symmetry and inhibits SHG. The figures are reproduced with the permission: (a), (b), (g), (h) ref. [153] © 2018 Wiley-VCH Verlag GmbH & Co. KGaA, Weinheim (c) ref. [156] © 2017 Nature Publishing Group (d), (e) ref. [113] © 2018 Nature Publishing Group (f) ref. [228] © 2015 Wiley-VCH Verlag GmbH & Co. KGaA, Weinheim (i) ref. [117] © 2017 Nature Publishing Group.

The FL-TMDs can display different symmetries even if they differ by one atomic layer. Since symmetry dictates the properties of the material, the role of layer dependence is essential to investigate. In 2013, Li et al. [62] probed the symmetry in FL 2H-MoS_2_ by using SHG. It is shown that the inversion symmetry is broken in the case of the odd number of layers and preserved in an even number of layers. Similar behavior in WS_2_ and WSe_2_ was reported by Zeng et al. [215]. As expected, they found significant SHG from the odd number of layers, while a negligible amount of SHG was detected from an even number of layers. In their measurement of the odd number of layers, the maximum SHG efficiency was reported in the monolayer while decreasing by increasing the odd number of layers. A similar gradual decrease of SHG with an increasing number of the odd number of layers was also reported in MoS_2_ [156]. Layer-dependent SHG was also investigated in an FL-MoTe_2_ [153]. The study reports the presence (absence) of the inversion symmetry for an even (odd) number of layers. It was also demonstrated that SHG intensity rises to maximum when the odd number of layers becomes five. This layer-dependent behavior in MoTe_2_ was attributed to the absorption offered for SHG by MoTe_2_ flakes. 1L-MoTe_2_ has a direct bandgap, whereas 3L-MoTe_2_ is an indirect bandgap material. So, the SHG absorption in the monolayer would be more significant than that in the trilayer resulting in efficient SHG in the case of the trilayer. When the layer number increases to 5, the extra number of layers minimizes the absorption, and hence the maximum SHG signal is achieved. As shown in Figure 8b, a further increase in the number of layers (7, 9) also decreases the SHG. It was stated that despite the fact that the absorption in thicker samples (layer number over 5) is weak, the total attenuation of the SH signal is high due to the more layers present. In contrast to SHG, THG magnitude does not depends on the inversion symmetry and presents in both even and odd number of layers. In ref. [156], a linear increase in THG magnitude with increased the number of MoS_2_ layers from 1 to 5 was reported. In all of these studies, HG was demonstrated to be an excellent tool to characterize the number of layers in TMDs (Table 4).

Our discussion focused mainly on the HG results from the 2H phase of the TMDs. Aside from this phase, there are 3R and 1Tʹ (semimetal monoclinic) phases of TMDs. Unlike 2H phase MoS_2_, the 3R MoS_2_ does not exhibit inversion symmetry in the case of even layers [153]. A phase match exists between the in-plane dipoles of each 3R phase layer, resulting in constructive interference of the SHG polarization in the thin-film limit below the coherence length [116]. Zhao et al. [154] investigated the layer and phase-dependent SHG from 2H and 3R MoS_2_ flakes. It was found that the SHG occurred at both even and odd numbers of layers in 3R MoS_2_, while it was negligible at even layers in 2H MoS_2_. It was also demonstrated that SHG in the 3R MoS_2_ scales quadratically with the number of layers up to six. The 3R MoS_2_ shows strong polarization dependence and more substantial (∼2 orders) SHG conversion efficiency than 2H MoS_2_. Song et al. [153] found a significant decrease of ∼2 orders in SHG magnitude when the 2H-MoTe_2_ phase is changed into the 1T′-MoTe_2_ phase. It is important to note that the energy difference between the two phases of MoTe_2_ is small. Moreover, contrary to the MoS_2_, the inversion symmetry is present (absent) in 1T′ and (2H) phases. Many investigations have been carried out to create phase transitions between two phases of the MoTe_2,_ such as laser irradiation and electrostatic doping. In one experiment [117] of electrostatic doping phase transition in MoTe_2_, the 2H phase indicated SHG without applying the gate voltage. However, the SHG decreased on applying voltage, indicating 2H to 1T′ phase change in the MoTe_2_ (Figure 8i).

### 5.4 Strain and edge induced HG

The exceptional strain limit of TMDs provides effective means of tuning electronic and optical properties [216]. This phenomenon leads to the potential application in strain modulated optoelectronic devices. Several investigations describe how strain can alter the optical properties of TMDs. For example, 10–15% biaxial tensile strain and 2% uniaxial tensile strain can produce semiconductor to metal and direct to indirect bandgap transition, respectively [217], [218], [219]. It also affects the Raman scattering [220], exciton phonon coupling [221], and single-photon emission [222] in TMDs. Similarly, strain should also modify the nonlinear response of the TMDs. Upon applied strain, the symmetry can be reduced, which is used to tune the SHG of TMDs [223]. For instance, Mennel et al. [223] reported the 2nd order photo-elastic tensors of WS_2_, MoS_2_, WSe_2_, and MoSe_2_ at 800 nm wavelength. The 2nd order photo-elastic tensors can be utilized to estimate the impact of strain on the SHG of material under test. Furthermore, it was shown that even small strains like those occurring upon polydimethylsiloxane (PDMS) exfoliation (0.2% strain) are sufficient to change SHG magnitude for 1L-TMDs noticeably. Liang et al. [224] measured the local strain in 1L-MoSe_2_ by polarization-resolved SHG. According to the study, strain changed the angle dependence and resulted in a 49% change in SHG intensity per 1% strain. In another study [225], polarization-resolved SHG was used to determine the folding angle and strain vector in FL-WS_2_ and found enhanced SHG by 1–9 times upon folding the 3L-WS_2_. This enhancement was attributed to the vector superposition of SHG wave vectors coming from the constituent layers of the fold with a 60° folding angle.

**Figure 9:**
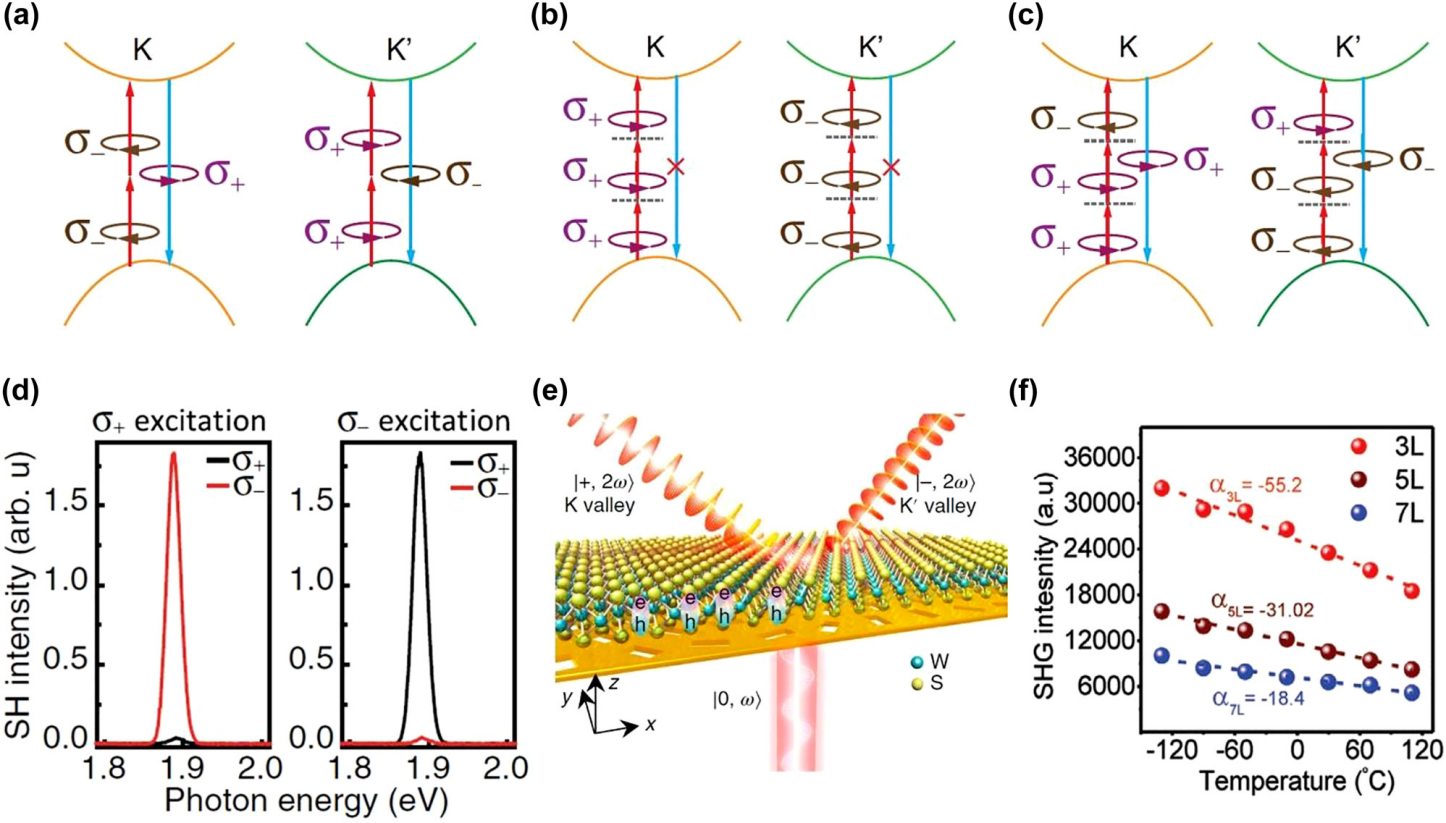
Valley and temperature assisted HG and selection rules for HG in TMDs. (a)–(c) Chiral selection rules of SHG and THG for TMDs. (d) Circularly polarization-resolved SHG spectra from 1L-WSe_2_ with the circularly polarized fundamental wave at 0.94 eV. (e) Schematic of Au-WS_2_ metasurface used for coherent steering of nonlinear chiral valley photons in 1L-WS_2_. (f) Temperature-dependent SHG spectra for MoSe_2_ flakes with different thicknesses. The figures are reproduced with the permission: (a)–(d) ref. [233] © 2019 Optical Society of America (e) ref. [111] © 2019 Nature Publishing Group (f) ref. [239] © 2020 Wiley-VCH Verlag GmbH & Co. KGaA, Weinheim.

Among harmonic orders, SHG was utilized widely to characterize the strain in TMDs [113, 226], but in 2019, THG was also employed to monitor the strain in WS_2_ [227]. Liang et al. [227] reported that THG can serve as a universal tool for all 2D materials without facing any symmetric constraint. The versatility of the THG characterization was tested successfully by applying it on the non-centrosymmetric 1L-WS_2_ and 2H 2L-WS_2_. THG was demonstrated as the most versatile method for monitoring tensile properties in any 2D material, suggesting its potential application in strained-induced nanophotonic devices.

Translational symmetry is broken at the edges of 2D crystals, leading to a change in the electronic bandgap compared to the central region [150]. This may result in different wavelength resonances compared to the central area and change the SHG at the edges of the 2D crystals. Lin et al. [77] found a strong SHG at the edges in pyramid-like WS_2_ compared to the central region of the WS_2_, Figure 4g. Yin et al. [150] employed SHG to detect the 1D edges and grain boundaries in 1L-MoS_2_ and found that SHG was significantly suppressed at the edges due to the destructive interference of the nonlinear waves produced from the neighboring grains with different orientations. Similarly, Cheng et al. [228] utilized SHG to retrieve the relative phase information to identify the crystalline orientation and edge termination of CVD grown 1L-MoS_2_. Lin et al. [229] demonstrated that it is possible to differentiate between the S-zigzag edge and S-Mo Klein edge (bare Mo atoms protruding from an S-zigzag edge) by SHG imaging. Apart from the SHG, THG can also have the ability to detect the edge states in CVD grown MoS_2_ by building the high contrast associated with grain boundaries after treating the MoS_2_ with common solvents usually used in the transfer process of two-dimensional materials [149]. The value of *χ* ^3^ was increased from 1.2 × 10^−19^ m^2^ V^−2^ to a value at the grain (3 × 10^−19^ m^2^ V^−2^) and grain boundaries (3.7 × 10^−19^ m^2^ V^−2^).

### 5.5 Valley dependent SHG

Valley polarization is one of the most important ways to control PL from TMDs, governed by the valley selection rule [230]. Optical selection rules establish a fundamental principle of permissible and prohibited transitions. Several studies [231, 232] have shown that +*σ* (−*σ*) light can only be absorbed and emitted at *K* (*K*′) valleys of the Brillouin zone in a one-photon case. In contrast to the linear optical selection rules that pertain to transitions between ground and excited states, nonlinear processes, such as optical HG, involve virtual excited states, which leads to more complex selection rules (Figure 9a–d). According to Seyler et al. [67], −*σ* (+*σ*) excitation photons are absorbed in 1L-WSe_2_ and lead to the emission of +*σ* (−*σ*) SHG photons. Similarly, Xiao et al. [230] investigated the nonlinear optical selection rules for SHG in 1L-WS_2_ based on the spin-valley locking relationship. It was also observed that compared with non-resonant excitation, the magnitude of the SHG was almost equal to unity under 1s excitation. An increase in helicity of −99% was achieved by pumping +*σ* light of 1.045 eV energy onto the 1L-WS_2_. In addition to the nonlinear selection rules for SHG, Cheng et al. [233] proposed nonlinear selection rules for THG in 2019. As shown in Figure 9c, none of the three +*σ* (−*σ*) photons can lead to the −*σ* (+*σ*) THG photon. The allowed transition can only happen when a pair of +*σ* (−*σ*) combines with one photon with helicity −*σ* (+*σ*) in order to generate the +*σ* (−*σ*) THG photon. It is also noticeable that linearly polarized light exhibits significantly higher THG than circularly polarized light.

In linear spectroscopy, the valley excitons are weak and extremely difficult to exploit for practical purposes. It is possible to attain a significant degree of valley polarization, but this is usually only possible at cryogenic temperatures. On the other hand, nonlinear valley emission, such as SHG, can be performed at room temperature and can achieve a 100% degree of valley polarization, suggesting a particular interest in valleytronic applications. On various staked TMDs, circular polarization-dependent SHG experiments were carried out (Table 4). It was found that the interlayer coupling does not significantly affect the valley-polarized SHG when a spiral or twisted angle 2L-TMDs is used [233]. The valley-polarization of 2H-MoSe_2_ was investigated using SHG. It was demonstrated that the valley imbalance can be modulated by changing the polarization state of the laser from linear to circular. Integrating the WS_2_ metasurface with a plasmonic metasurface enabled Hu et al. [111] to steer SHG valley photons in any desired direction (Figure 9e). A unique approach to manage nonlinear valley-locked twisted vortex emissions has been presented by Yang et al. [234]. The study is based on pumping a monolayer WS_2_ using a vector beam. The SHG photons are emitted from both *K* and *K*′ valleys from monolayer WS_2_ bearing separate optical vortices, which leads in the formation of SHG vector beams. Conical refractions with topological charge “1/2” were employed to create first-order vector beams including radially and azimuthally polarized beams, as well as complete Poincaré beams.

### 5.6 Controlling HG by some other factors

Many other approaches exist for enhancing or controlling HG in TMDs. By optically tuning the density of photocarriers in 1L-TMDs, one can achieve ultrafast modulation of *χ* ^(2)^. It was found that by depopulating the conduction band electrons at the vicinity of the high-symmetry *K*/*K*′ points of MoS_2_, the impact of interband electronic transitions in the effective *χ* ^(2)^ of the 1L-MoS_2_ can be suppressed, permitting the all-optical modulation of the SHG [235]. This study demonstrates that the SHG can be modulated by up to 55% within 250 fs due to photo carriers. The work [235] reveals that SHG can be used as a novel method for studying photo-carrier dynamics in TMDs.

The optoelectronic properties TMDs such as carrier mobility [236], phonon modes [237], and band gap [238] can be considerably altered by thermal variations. Because structural symmetry affects the SHG, it is possible to use SHG to examine the behavior of temperature variation in 1L-TMDs. Thermal variations have a significant influence on the layer-dependent SHG. Khan et al. [239] found that the 1L-MoSe_2_ showed an increase in SHG (25.8%) with temperature increase, but MoSe_2_ 3L, 5L, and 7L showed considerable decreases of 55.2, 31.02, and 18.4%, respectively Figure 9f. Other TMDs, such as WS_2_, MoS_2_, and WSe_2,_ also showed a similar structural trend. SHG measurements on the 1L-WSe_2_ revealed other exciting effects associated with HG in TMDs, including quantum interferences. Verre et al. [240] introduced the concept of TMD nanoantennas, which allows strong coupling between geometrical optical modes and excitons within the same nanophotonic object. Later, Busschaert et al. [203] fabricated patterned WS_2_ disks that exhibit strong enhancements of SHG in the visible range due to their high internal resonant electric fields. The implementation of these SHG enhanced TMDs into resonator arrays represents a significant opportunity for the development of highly efficient nonlinear TMD meta-surfaces that can be used for many nonlinear photonic devices. It is exciting to think that there might yet be exciting new physical phenomena concerning nonlinear polarization in TMDs due to the rapid expansion of HG in TMDs.

## 6 Conclusion and future perspectives

This review provides a brief overview of research studies conducted in HG of TMDs in the past and present. Due to their superior nonlinearity and phase matching, TMDs based HG has attracted significant attention to their many applications in next-generation optoelectronic devices. A review of the progress made in the HG investigation of TMDs has been presented, ranging from simple layered TMDs to stacked/twisted heterostructures. In addition, we discussed how the nanostructures interacting with the TMDs can enhance or modulate the HG. We have finally explored the HG engineering in TMDs using various techniques, such as electrical gating, excitonic effect, mechanical tuning, valley-assisted HG, and many others. By providing a comprehensive account of the field, we hope to aid readers in establishing a global perspective, offer access to recent research developments, and define future research directions related to this field.

Although HG has brought immense advantages to nonlinear optics, there are still challenges associated with the study of HG in TMDs. The production of high-quality 2D films is one of the main challenges. As a result of structural changes in the material during the fabrication process, TMDs are susceptible to various problems, including surface defects, strain, vacancy, doping, and dangling bonds. In order to improve the quality and size of the films, existing fabrication processes should be improved. In particular, techniques such as Au-based exfoliation and parametric optimization of the CVD process have been demonstrated to enhance the size and quality of flakes. This review aimed to provide a comprehensive overview of the experimental outcome of HG in the TMDs. However, it would be beneficial if more efforts were devoted to investigating the theoretical implications of HG in TMDs. For instance, substrate effects are particularly critical when it comes to experiments with atomically thin TMDs. So, it remains necessary to develop a quantitatively accurate model of the HG processes in TMDs to construct a framework suitable for understanding the underlying physics. Patterned TMD nanostructures can significantly enhance HG. Therefore, research on HG using these patterned TMD nanostructures is highly desirable. The combination of biomolecules and TMDs and their role in HG is unexplored. One of the properties of biological macromolecules such as proteins is that they usually have broken inversion symmetry and can trigger HG. With advancements in THz research of TMDs [32], investigations on their nonlinear performance should follow logically. Additionally, there is a lack of research on HHG investigations of TMDs and their heterostructure. The tuning parameters presented in this study were used to control SHG and THG in TMDs; however, how these tuning factors may be used to control HHG in TMDs has not been examined yet. As a result, a thorough analysis of these HHG investigations is essential. Additionally, since multiphoton processes display distinct optical selection criteria from linear optical responses, SHG offers a novel platform for investigating fascinating phenomena such as dark excitons and valley degree of freedom [207, 230, 233]. When combined with significant exciton effects, SHG enables the investigation of unusual exciton states, such as the excited states of exciton polaritons recently described in WS_2_ [243]. Hence, if these challenges can be adequately overcome, it is expected that this new field will significantly impact emerging technologies and fundamental science.
